# Smoking-Associated Enrichment of CXCL13^+^ Tfh Cells and VCAM1^+^ Fibroblasts Is Linked to Tertiary Lymphoid Structure Formation in Lung Adenocarcinoma

**DOI:** 10.7150/jca.138884

**Published:** 2026-09-03

**Authors:** Yong Jun Choi, Chi Young Kim, Eun Young Kim, Sang Hoon Lee, Min Kyung Park, Yoon Soo Chang

**Affiliations:** 1 Division of Pulmonary and Critical Care Medicine, Department of Internal Medicine, Gangnam Severance Hospital, Yonsei University College of Medicine, Seoul, Republic of Korea.; 2 Division of Pulmonary and Critical Care Medicine, Department of Internal Medicine, Severance Hospital, Yonsei University College of Medicine, Seoul, Republic of Korea.

**Keywords:** cancer-associated fibroblast, lung adenocarcinoma, tumor microenvironment, Tfh cells, CXCL13, tertiary lymphoid structure

## Abstract

**Background:**

Smoking history is associated with improved immune checkpoint inhibitor treatment responses. We investigated smoking-associated differences in the tumor microenvironment (TME) of lung adenocarcinoma (LUAD) and identified biomarkers associated with tertiary lymphoid structure (TLS) formation.

**Methods:**

Single-cell RNA-sequencing datasets from tumors and matched adjacent normal tissues from five current smokers and five never-smokers with LUAD were integrated, and the T- and NK-cell compartments were analyzed in detail. Key findings were further evaluated using public LUAD scRNA-seq datasets and validated by immunofluorescence in formalin-fixed paraffin-embedded LUAD tissues using QuPath-based image analysis.

**Results:**

In current smokers, follicular helper T (Tfh) cells were enriched in the TME, whereas CD4⁺ tissue-resident memory T cells were reduced (R_O/E_, median [interquartile range (IQR)]: 2.1 [1.5-5.7] vs. 1.0 [0.5-1.5], p=0.040; and 0.7 [0.6-1.1] vs. 1.7 [1.6-2.4], p<0.001, respectively). The Tfh cluster showed high expression of *CXCL13* and increased expression of* PDCD1* and *CTLA4*. Immunofluorescence confirmed increased CXCL13⁺CD4⁺ cells in current smokers (83.0 [10.0-218.5] vs. 10.0 [2.0-60.5] cells/0.25 mm², p<0.001). Gene set analysis showed enrichment of a B-cell chemotaxis signature in the Tfh cluster (normalized enrichment score, 1.693; adjusted p=0.023), and CXCL13⁺CD4⁺ T-cell density correlated with CD20⁺ B-cell density (Spearman ρ=0.445, p<0.001). Current smokers also showed greater TLS burden, with higher TLS counts and larger TLS areas, and had increased VCAM1⁺ fibroblast abundance (283.0 [118.5-580.5] vs. 98.5 [32.5-191.0] cells/0.25 mm², p<0.001), which strongly correlated with CD20⁺ cell density (Spearman ρ=0.766, p<0.001).

**Conclusions:**

Smoking-associated enrichment of CXCL13⁺CD4⁺ Tfh cells was associated with B-cell accumulation and TLS formation in LUAD. These findings identify the CXCL13⁺ Tfh-B-cell axis, together with VCAM1⁺ fibroblast-rich stromal niches, as potential biomarkers and therapeutic targets in LUAD.

## Introduction

Lung cancer is one of the leading causes of cancer-related mortality worldwide, with an estimated 2 million diagnoses and 1.8 million deaths 1,2. Smoking plays a pivotal role in the initiation and progression of lung adenocarcinoma (LUAD) and squamous cell carcinoma 3,4. A single cigarette contains over 7000 toxic compounds and 60 carcinogens, including polycyclic aromatic hydrocarbon carcinogens such as benzo[a]pyrene and nicotine-derived nitrosamine ketone 4.

Recent trends in lung cancer therapy have shifted toward immune checkpoint inhibitors (ICIs) targeting programmed cell death protein 1 (PD-1), programmed death-ligand 1 (PD-L1), and cytotoxic T-lymphocyte-associated protein 4 (CTLA-4), demonstrating promising efficacy in treating metastatic non-small cell lung cancer (NSCLC) 5-7. These agents have demonstrated clinical activity, particularly in patients with high PD-L1 expression, and have been associated with improved overall survival 8. However, the predictive performance of PD-L1 as a biomarker remains limited, because a substantial proportion of patients with high PD-L1 expression fail to respond to ICIs 9,10. In addition to PD-L1, other potential biomarkers, including tumor mutational burden and features of the tumor immune microenvironment, have been investigated to predict ICI responses 11. However, more robust biomarkers are required to accurately predict the response to ICIs and improve patient selection for immunotherapy.

Despite the well-established harmful effects of smoking, several studies have highlighted enhanced efficacy of ICIs in smokers 12,13. In particular, higher densities of tumor- and stromal-infiltrating CD4⁺ and CD8⁺ T cells were observed in smokers, suggesting a more active antitumor immune response 14. Recent studies have demonstrated that T cells can actively promote the formation of tertiary lymphoid structures (TLS), which are associated with improved antitumor immunity 15,16. TLS-rich tumor microenvironments exhibit organized lymphoid aggregates with enhanced local antigen presentation and coordinated adaptive immune responses, leading to more effective tumor control. However, the precise mechanisms underlying enhanced responsiveness to ICIs in smokers remain unclear.

This study aimed to investigate the biological mechanisms underlying the favorable responsiveness to ICIs associated with smoking by comparing T-cell and NK-cell profiles in the tumor microenvironment (TME) between current smokers and never-smokers. We analyzed single-cell RNA sequencing (scRNA-seq) data from tumor and matched adjacent normal lung tissues obtained from patients with early-stage LUAD who underwent surgical resection.

## Methods

### Study population and patient recruitment

**Figure 1:**
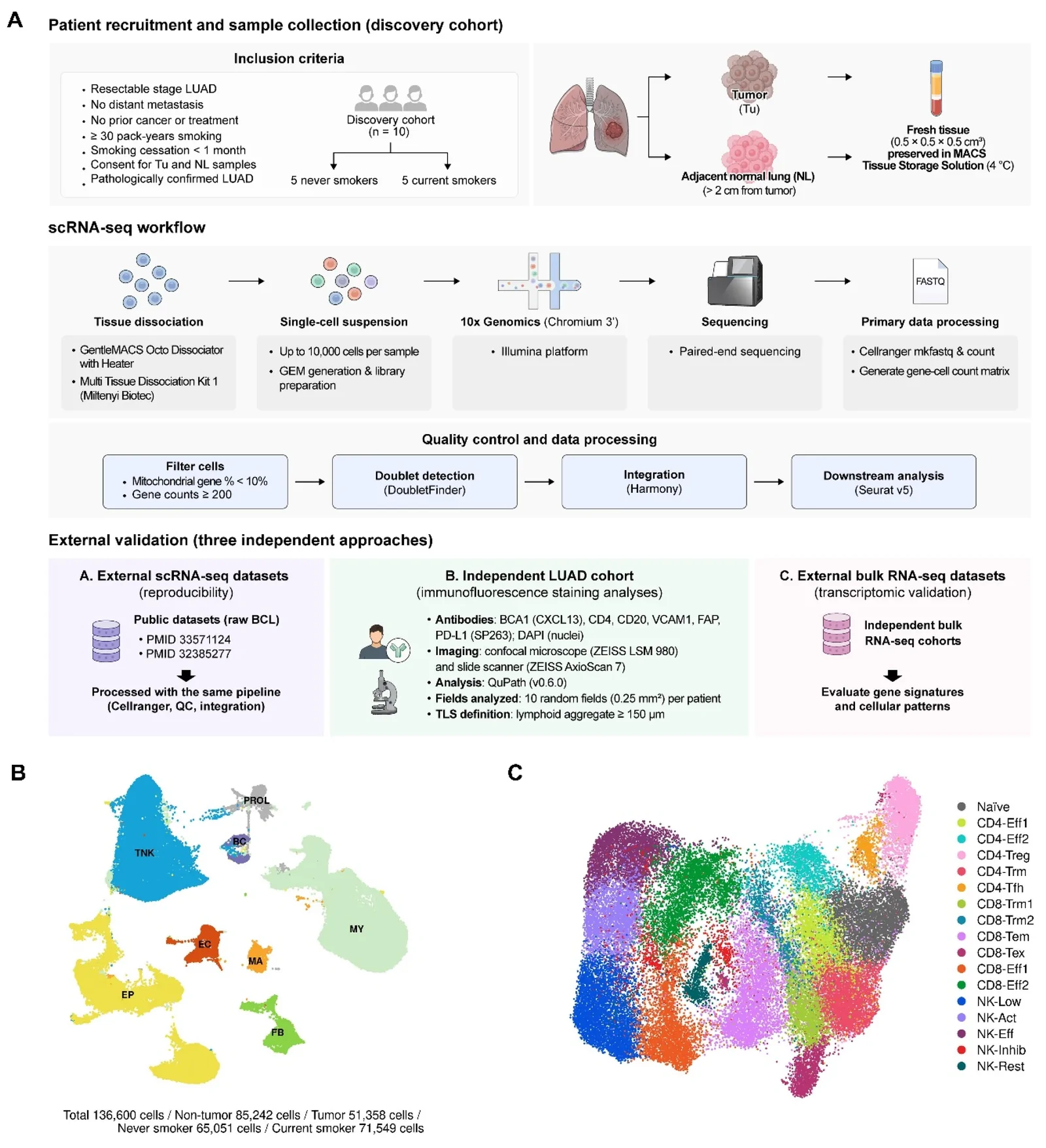
** Single-cell landscape of T and NK cells in lung cancer.** (A) Overview of sequencing datasets, including sample sources, preprocessing workflow, and distribution across cohorts. (B) UMAP visualization of major cell lineages. (C) UMAP visualization of all T and NK cells.

Samples were collected from patients who underwent curative surgical resection for LUAD at Gangnam Severance Hospital, Yonsei University College of Medicine, between 2019 and 2022 (Fig. 1A) 17-19. The discovery cohort consisted of ten patients with LUAD, including five current smokers and five never-smokers. Current smokers were defined as patients with a smoking history of at least 30 pack-years who had continued smoking until LUAD diagnosis and had stopped smoking for less than one month before surgery. Never-smokers were defined as those who had never smoked; former smokers were excluded to minimize biological heterogeneity associated with variable smoking cessation durations and cessation-related changes in the tumor microenvironment. Eligible patients met the following criteria: (1) surgically resectable stage LUAD; (2) no evidence of distant metastasis on staging workup, including positron emission tomography-computed tomography and brain magnetic resonance imaging; (3) no history of malignancy or anticancer treatment; (4) availability of residual tumor (Tu) and adjacent normal lung (NL) tissue for analysis; and (5) pathological confirmation of LUAD. Patients who had received neoadjuvant or adjuvant chemotherapy were excluded. This cohort was used as the discovery cohort for the subsequent analyses.

### Tissue processing, scRNA-seq library construction, and quality control

Paired tumor and adjacent NL tissues were obtained from ten patients in the discovery cohort. Intraoperative specimens were transferred to the pathology laboratory, where frozen section examinations were performed to confirm LUAD. Fresh tumor tissue and adjacent NL tissue collected more than 2 cm from the tumor margin were cut into approximately 0.5 × 0.5 × 0.5 cm³ pieces and processed by a pathologist. Samples were preserved in MACS Tissue Storage Solution at 4 °C and sent to Macrogen Korea (Seoul, Republic of Korea) for downstream processing according to the Chromium Single Cell 3′ Gene Expression protocol (10x Genomics). Single-cell suspensions were prepared using a gentleMACS Octo Dissociator with a Heater and a Multi-Tissue Dissociation Kit 1 (Miltenyi Biotec). Libraries were generated by loading up to 10,000 cells per sample using a Chromium Single Cell 3′ GEM Library and Gel Bead Kit v3. After polyadenylated mRNA capture with poly(dT) primers, barcoded full-length cDNA was synthesized and paired-end sequencing was performed on an Illumina platform. Raw base-call files were demultiplexed into FASTQ files using Cell Ranger mkfastq and counted. Quality control was performed using Seurat (v5.0.1), excluding cells with > 10% mitochondrial gene content or < 200 genes. Expected doublets were identified and removed from each sample using DoubletFinder (v2.0.4). After quality control and doublet removal, the scRNA-seq data from all samples were integrated using the Harmony algorithm to minimize batch effects.

### External validation cohorts

Three independent external validation approaches were used to assess the robustness of the findings. First, for transcriptomic validation, we analyzed publicly available LUAD scRNA-seq datasets comprising four never-smokers and three current smokers from previously published studies 20,21. These datasets were processed from raw FASTQ files using the same preprocessing and downstream analytical workflow as the discovery cohort, including read alignment, quality control, data integration, clustering, and cell-type annotation. Second, for spatial validation, an independent cohort of formalin-fixed, paraffin-embedded LUAD tissues was used for the immunofluorescence-based evaluation of key cellular markers and tissue architecture. Third, for external transcriptomic validation at the bulk level, publicly available LUAD RNA-sequencing cohorts were analyzed to examine whether the key gene expression patterns identified in the discovery analyses were recapitulated in larger patient cohorts from cBioPortal/GDC, including the CPTAC GDC 2025, OncoSG 2020, and TCGA GDC cohorts 22-24. Kaplan-Meier survival analysis was performed separately using the TCGA GDC cohort.

### Immunofluorescence staining and image analysis

Immunofluorescence (IF) staining was performed using formalin-fixed, paraffin-embedded LUAD tissues from an independent validation cohort approved by IRB No. 3-2024-0456. The following primary antibodies were used: anti-CXCL13/BCA1 (GeneTex, GTX108471), anti-CD4 (Ventana, 790-4423), anti-CD20 (Ventana, 760-2531), anti-VCAM1 (Abcam, ab134047), anti-FAP (Invitrogen, PA5-99458), and anti-PD-L1 (Ventana, 790-4905). Nuclei were counterstained with DAPI using ProLong™ Gold Antifade Mountant (Thermo Fisher Scientific, P36931). Fluorescence images were acquired using a confocal microscope (ZEISS LSM 980; ZEISS, Oberkochen, Germany) and a whole-slide scanner (ZEISS Axioscan 7; ZEISS). Digital image analysis was performed using QuPath software (version 0.6.0). Cells were first detected using the Cell Detection function, after which positive cells were classified using the Create Single Measurement Classifier function according to marker intensity. The co-expression of multiple markers was then determined using the Create Composite Classifier function. For quantitative analysis, ten randomly selected fields per patient (0.25 mm² per field) were evaluated. A TLS was defined as a dense nodular aggregate of lymphoid cells resembling a lymphoid structure, with or without a germinal center, and with a minimum diameter of 150 μm 15,25.

### Bioinformatic and statistical analysis

All downstream single-cell analyses were performed using Seurat R package (version 5.0.1). Cell clustering was performed using the FindNeighbors and FindClusters functions, and marker genes were identified using the FindAllMarkers and FindMarkers functions. Cell-type annotation was based on the expression of canonical marker genes and previously reported markers. Ligand-receptor interaction analysis was performed using CellChat (version 1.6.1) and gene set enrichment analysis was conducted using clusterProfiler (version 4.8.3). Adjusted p-values for gene set enrichment analysis were calculated using the Benjamini-Hochberg method.

To compare cluster distribution according to smoking status, the ratio of observed to expected counts (R_O/E_) was calculated for each T/NK-cell subcluster in each sample based on the chi-square-derived observed and expected counts. Group-wise differences in R_O/E_ values and other continuous variables between current smokers and never-smokers were evaluated using the Wilcoxon rank-sum test unless otherwise specified. Correlations between cellular density and gene expression levels were assessed using Spearman's rank correlation analysis.

Trajectory and pseudotime analyses of CD4⁺ T-cell subsets were performed using the R packages Slingshot (v2.18.0) and condiments (v1.18.0). Differences in the pseudotime distributions between groups were assessed using the Wilcoxon rank-sum test. For external bulk RNA-seq validation, gene expression values were z-score-normalized and patients were stratified into low- and high-expression groups (Q1 vs. Q4) for Kaplan-Meier survival analysis. Differences in survival were compared using log-rank tests. All statistical tests were two-sided, and p<0.05 was considered statistically significant.

### Ethics

This study was approved by the Institutional Review Board of our institution (IRB No. 3-2019-0299). The acquisition of patient specimens for external validation was approved by a separate protocol (IRB No. 3-2024-0456). Written informed consent was obtained from all the participants.

## Results

### Data integration and annotation

To characterize the T- and NK-cell compartments in the TME of LUAD, tumors and matched adjacent normal tissues were obtained from five current smokers and five never-smokers (Fig. 1A). After quality control, scRNA-seq data from these samples were integrated using the Harmony algorithm 26. For validation, we integrated publicly available scRNA-seq datasets from patients with non-advanced-stage LUAD with available smoking history data, including four never-smokers and three current smokers. The discovery dataset revealed eight major cell lineages defined by the expression of canonical marker genes. The major cell lineages identified in the discovery dataset include T/NK cells, B cells, myeloid cells, epithelial cells, endothelial cells, fibroblasts, mast cells, and proliferative cells (Fig. 1B). Subsequently, the discovery dataset was used as a reference to classify cell types in the validation dataset (Supplementary Fig. s1A and B). The baseline characteristics of the discovery dataset are presented in Supplementary Tables s1 and s2. No statistically significant differences were observed between current smokers and never-smokers in age, TNM stage, or *EGFR* mutation status. However, sex distribution differed significantly between the groups: all five current smokers were male, whereas four of the five never-smokers were female (p=0.048).

After quality control, 136,600 cells were retained for the discovery dataset, and 52,443 cells were retained for the validation dataset (Supplementary Table s3). The T/NK-cell compartments were extracted from both datasets for downstream analysis, yielding 45,538 cells in the discovery dataset and 23,306 cells in the validation dataset (Supplementary Table s4). After excluding putative alveolar-cell and B-cell contaminating clusters, 43,328 cells in the discovery dataset and 21,075 cells in the validation dataset were retained for subsequent T/NK-cell subtype analyses (Supplementary Table s5). To confirm annotation consistency, we compared our cell identities with the Azimuth Lung reference atlas v2.0 (Supplementary Fig. s1C) 27. The proportions of major cell lineages in the discovery and validation datasets are summarized in Supplementary Table s4.

### CXCL13⁺ T follicular helper (Tfh) cells are enriched in the TME of current smokers

**Figure 2:**
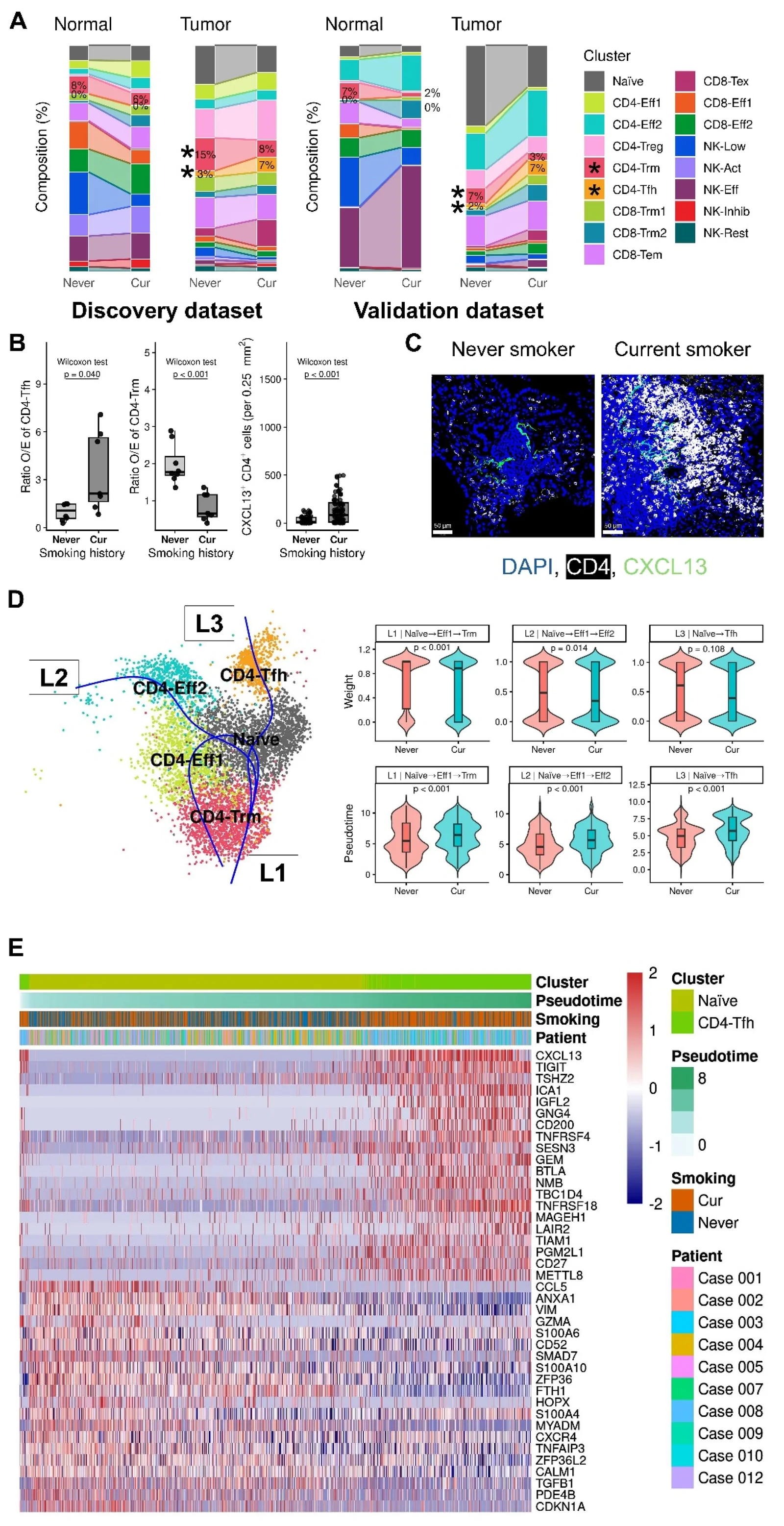
** Expansion of CXCL13⁺CD4⁺ Tfh cells in smokers.** (A) Proportional composition of T/NK-cell clusters stratified by smoking status in normal and tumor tissues across cohorts. Asterisks (*) indicate clusters showing significant differences in both cell proportion and ratio of observed to expected (R_O/E_) values between groups. In the tumor tissues of both the discovery and validation sets, CD4-Trm cells were less abundant in current smokers, whereas CD4-Tfh cells were more abundant in current smokers. (B) Comparison of CD4-Tfh and CD4-Trm cell enrichment between the smoking groups. Single-cell RNA sequencing-derived R_O/E_ values are shown alongside validation using an independent cohort, where CXCL13⁺CD4⁺ cell abundance was quantified by immunofluorescence. Current smokers showed increased enrichment of CD4-Tfh cells and decreased enrichment of CD4-Trm cells compared to never-smokers. Consistently, CXCL13⁺CD4⁺ cells were more abundant in smokers by immunofluorescence analysis. p-values were calculated using the Wilcoxon rank-sum test. (C) Representative immunofluorescence images showing CXCL13⁺CD4⁺ T cells (CXCL13, green; CD4, white; DAPI, blue) in the tumor tissues. Scale bar: 50 µm. (D) Trajectory inference analysis of CD4⁺ T cells showing differentiation from the naïve to effector and Tfh states. Distinct lineage paths (L1-L3) are indicated, with comparisons of trajectory weights and pseudotimes between smoking groups. (E) Heatmap of the gene expression dynamics along the naïve-to-Tfh trajectory ordered by pseudotime. Cells were annotated based on cluster identity, smoking status, and patient.

The T/NK compartment was resolved into 17 subclusters based on canonical marker gene expression (Fig. 1C and 2A, Supplementary Fig. s2A and B, and Supplementary Table s5). To compare differences in cluster distribution according to smoking status, the ratio of observed to expected counts (R_O/E_) was calculated for each T/NK subcluster in each sample using the chi-square test. In the TME of current smokers, the CD4⁺ Tfh cluster was significantly enriched, whereas the CD4⁺ Trm cluster was markedly reduced (current smoker vs. never-smoker, CD4^+^ Tfh: R_O/E,_ median [interquartile range (IQR)], 2.11 [1.53-5.68] vs. 1.04 [0.53-1.48], p=0.040; CD4⁺ Trm: 0.66 [0.56-1.10] vs. 1.75 [1.64-2.37], p<0.001; Fig. 2A, B; Supplementary Fig. s3). A similar compositional trend was observed in the validation dataset. No substantial differences were observed in the proportions of the remaining T-cell or NK-cell subclusters between current smokers and never-smokers. The CD4⁺ Tfh cluster showed increased expression of multiple activation-associated markers, including *CD2, CD6, CD82, TNFRSF4* (OX40)*, TNFRSF18* (GITR)*, ICOS, CD27,* and *CD28*, and was characterized by prominent *CXCL13* expression (Supplementary Fig. s2B). Consistent with these transcriptomic findings, IF analysis of an independent patient cohort demonstrated a significantly higher abundance of CXCL13⁺CD4⁺ T cells in tumors from current smokers than in those from never-smokers (83.00 [10.00-218.50] vs. 10.00 [2.00-60.50] cells per 0.25 mm², p<0.001; Fig. 2B, C). CD4⁺ Tfh cells also exhibited relatively high expression of immune checkpoint-related molecules, including* PDCD1* (PD-1) and *CTLA4* (CTLA-4) (Supplementary Fig. s2B). Consistent with these findings, the external RNA-seq validation cohort revealed positive correlations between *CXCL13* expression and *PDCD1* and *CTLA4* across cohorts, and with* CD274 (PD-L1*) in some of the external datasets (Supplementary Fig. s4) 22-24. In addition, IF staining of LUAD samples demonstrated a positive correlation between CXCL13⁺CD4⁺ T cells and PD-L1 expression **(**Supplementary Fig. s4B-D).

Trajectory analysis of CD4⁺ T cells identified three major differentiation paths originating from naïve cells and leading to CD4⁺ Eff, CD4⁺ Trm, and CD4⁺ Tfh states (Fig. 2D). Within the naïve-to-Tfh trajectory, cells from current smokers occupied later pseudotime positions than those from never-smokers (5.9 [4.4-7.8] vs. 5.0 [3.4-7.1], p<0.001). Along this inferred trajectory, the cells progressively upregulated *CXCL13, TIGIT, TSHZ2, ICA1*, and *IGFL2*, consistent with the acquisition of a Tfh-like activated and immune regulatory program, whereas *CCL5, ANXA1, VIM, GZMA*, and *CD52* were downregulated, indicating the attenuation of cytotoxic and migratory features (Fig. 2E).

### CD4⁺ Tfh cell enrichment in the TME is associated with B-cell accumulation and TLS development

**Figure 3:**
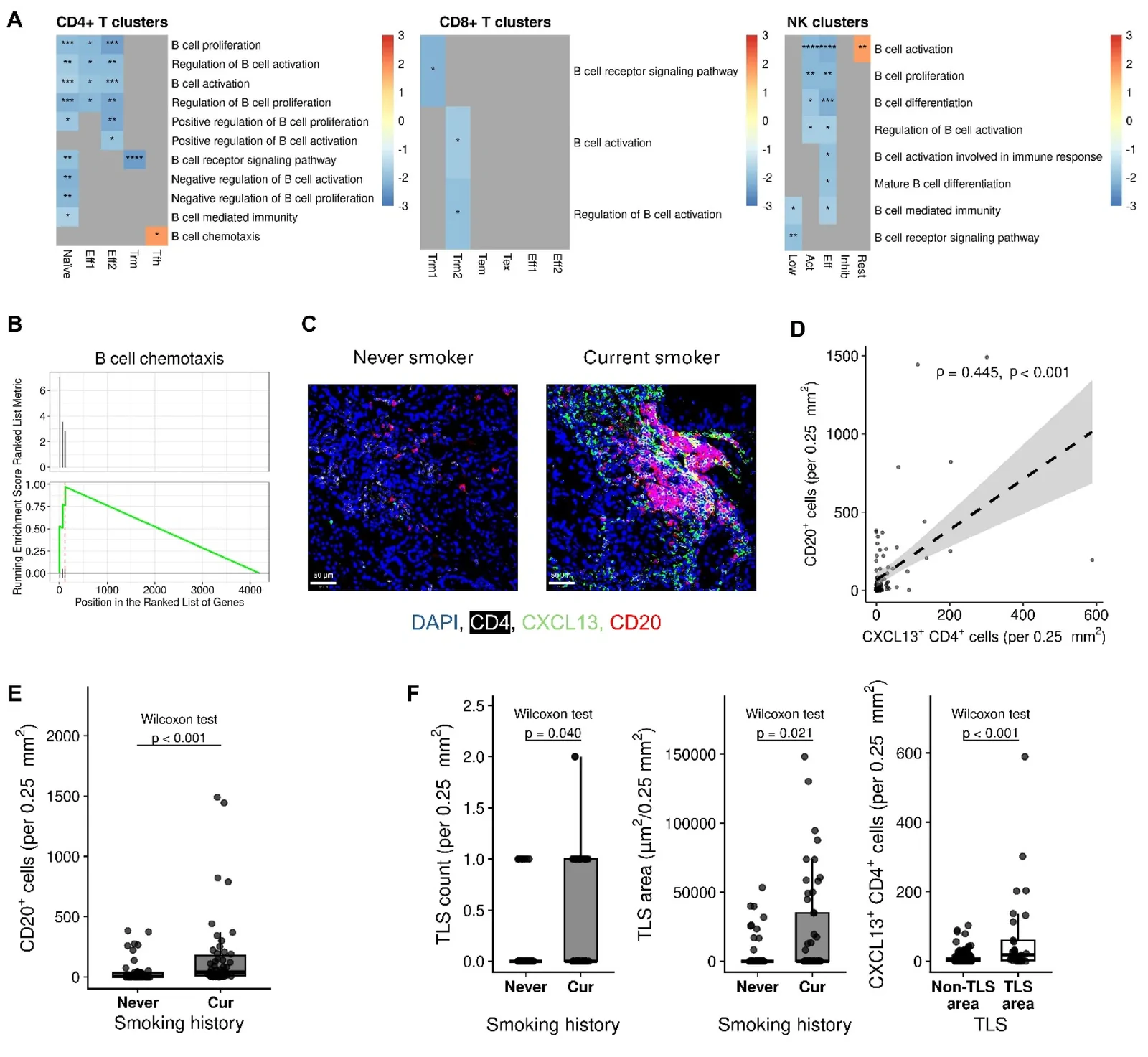
** Smoking-associated enrichment of CXCL13⁺CD4⁺ T cells and B-cell recruitment with tertiary lymphoid structure (TLS) formation.** (A) Gene set enrichment analysis (GSEA) of B-cell-related pathways across CD4⁺, CD8⁺, and NK-cell clusters. B-cell activation, proliferation, and receptor signaling pathways were predominantly enriched in specific CD4⁺ T-cell clusters. (B) GSEA plot demonstrating the enrichment of the B-cell chemotaxis gene set in CD4⁺ Tfh cells. (C) Representative multiplex immunofluorescence images showing DAPI (blue), CD4 (white), CXCL13 (green), and CD20 (red). Co-localization of CXCL13⁺CD4⁺ T cells with CD20⁺ B cells is observed in tumor regions. Scale bar: 50 µm. (D) Correlation between CXCL13⁺CD4⁺ T cells and CD20⁺ B cells, indicating a positive association. (E) Comparison of CD20⁺ B-cell density according to smoking history. Current smokers showed significantly higher levels of CD20⁺ cell infiltration. (F) TLS-related analysis. Current smokers had increased TLS counts and larger TLS areas. CXCL13⁺CD4⁺ T cells were enriched in TLS regions compared with non-TLS regions.

Gene set enrichment analysis showed that the CD4⁺ Tfh cluster was preferentially enriched for B-cell-related programs, including B-cell proliferation, activation, receptor signaling, and chemotaxis (Fig. 3A; Supplementary Fig. s2C-E). Among these, the B-cell chemotaxis signature was significantly enriched (normalized enrichment score (NES) = 1.693, adjusted p=0.023; Fig. 3B).

Consistent with these transcriptomic findings, IF imaging demonstrated more prominent accumulation of CXCL13⁺CD4⁺ T cells and CD20⁺ cells in tumors from current smokers than in those from never-smokers (Fig. 3C). In addition, the number of CXCL13⁺CD4⁺ T cells was positively correlated with CD20⁺ cell density (Spearman ρ = 0.445, p<0.001; Fig. 3D), and CD20⁺ B-cell density was significantly higher in current smokers than in never-smokers (41.5 [9.0-190.0] vs. 5.0 [0.0-36.0] cells per 0.25 mm², p<0.001; Fig. 3E).

Current smokers also exhibited greater TLS burden than never-smokers, with significantly higher TLS counts and larger TLS areas on field-based quantification (0.0 [0.0-1.0] vs. 0.0 [0.0-0.0] per 0.25 mm², p=0.040; and 0.0 [0.0-35011.3] vs. 0.0 [0.0-0.0] μm² per 0.25 mm², p=0.021; Fig. 3F). Moreover, CXCL13⁺CD4⁺ T-cell density was significantly higher in TLS areas than in non-TLS areas (19.0 [3.0-60.0] vs. 3.0 [0.5-10.0] per 0.25 mm², p<0.001; Fig. 3F), further supporting the association of CXCL13⁺ Tfh cells with TLS development in the TME.

### VCAM1⁺ fibroblasts are associated with CXCL13⁺ Tfh enrichment and TLS development

**Figure 4:**
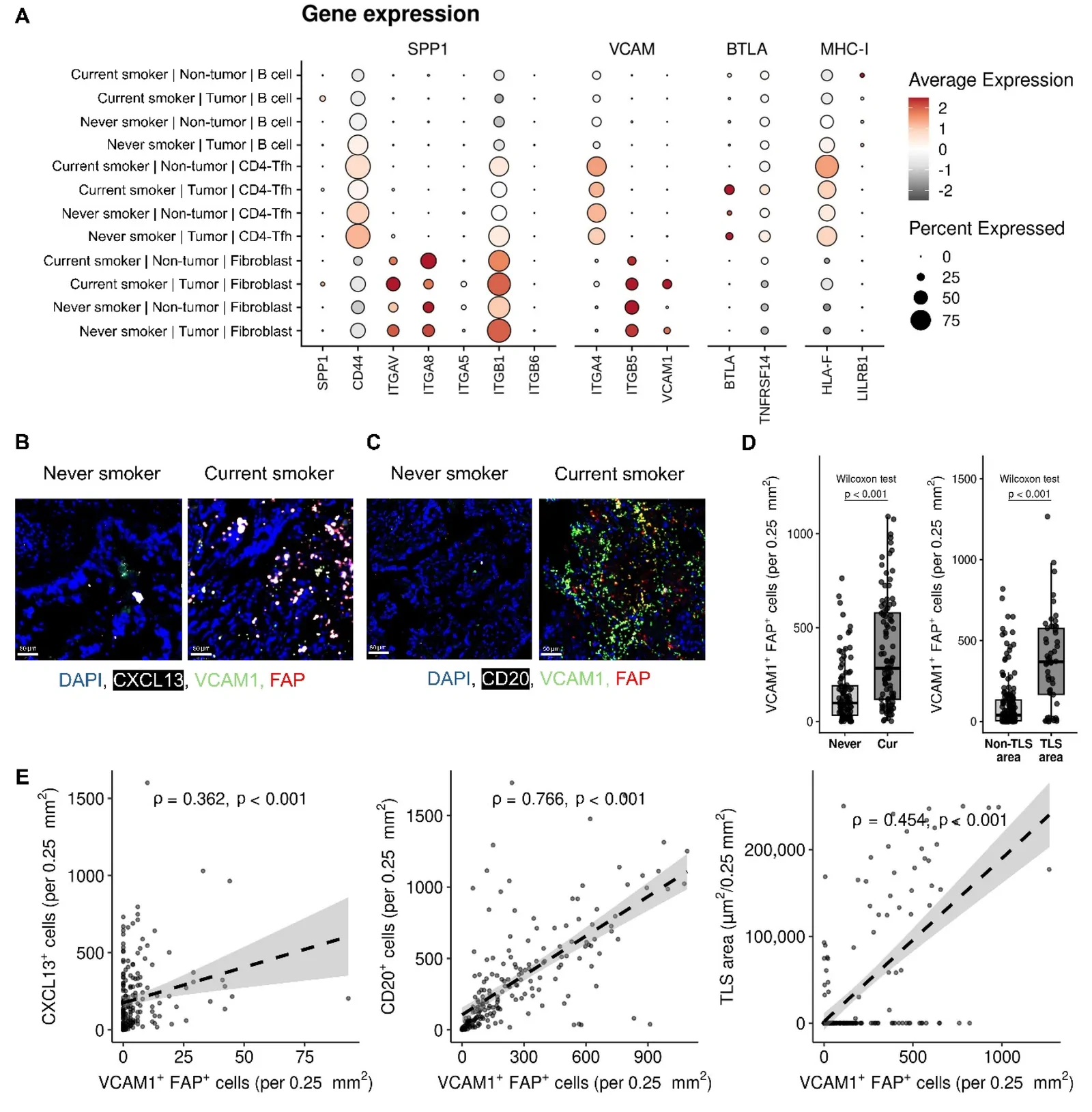
** VCAM1⁺FAP⁺ stromal cells are associated with CXCL13⁺CD4⁺ Tfh cells and B-cell infiltration in smokers.** (A) Dot plot showing the expression of ligand-receptor genes related to the SPP1, VCAM, BTLA, and MHC-I signaling pathways across major cell populations stratified by smoking status and tissue type. VCAM-related genes are predominantly expressed in stromal compartments, including fibroblasts. (B) Representative multiplex immunofluorescence images showing co-localization of CXCL13⁺ cells with VCAM1⁺FAP⁺ stromal cells. DAPI (blue), CXCL13 (white), VCAM1 (green), and FAP (red) staining. Scale bar: 50 µm. (C) Representative multiplex immunofluorescence images showing co-localization of CD20⁺ cells with VCAM1⁺FAP⁺ stromal cells. DAPI (blue), CD20 (white), VCAM1 (green), and FAP (red) staining. Scale bar: 50 µm. (D) Quantification of VCAM1⁺FAP⁺ stromal cell density based on multiplex immunofluorescence according to smoking history and TLS status. Current smokers exhibited significantly higher VCAM1⁺FAP⁺ cell infiltration than never-smokers, and VCAM1⁺FAP⁺ cells were enriched in TLS regions compared with non-TLS regions (Wilcoxon test, p<0.001 for both). (E) Correlation analyses showing positive associations of VCAM1⁺FAP⁺ stromal cells with CXCL13⁺ cells, CD20⁺ cells, and TLS area, as assessed by Spearman's correlation analysis.

To explore potential intercellular communication patterns associated with the enrichment of CD4⁺ Tfh cells, B cells, and TLS formation in tumors from current smokers, we performed CellChat-based ligand-receptor interaction analysis across TLS-related cell populations 28. Cells were stratified according to tissue type (adjacent normal vs. tumor) and smoking status (never-smoker vs. current smoker) in the discovery dataset. Across these subgroup analyses, 96 putative ligand-receptor interactions were inferred, with the SPP1, VCAM, BTLA, and MHC-I signaling axes showing relatively greater predicted activity in tumors from current smokers (Supplementary Fig. s5 and Fig. 4A). Among the analyzed cell types, fibroblasts showed the most prominent predicted outgoing signaling activity, indicating a fibroblast-centered communication pattern in the CellChat analysis (Supplementary Fig. s5).

Representative IF images of tumor tissues from ten never-smokers and ten current smokers showed more abundant VCAM1⁺FAP⁺ fibroblasts in current smokers, frequently localized within regions enriched in CXCL13⁺ or CD20⁺ cells (Fig. 4B, C). Quantitative analysis confirmed that VCAM1⁺FAP⁺ fibroblasts were significantly more abundant in current smokers than in never-smokers (283.0 [118.5-580.5] vs. 98.5 [32.5-191.0] cells per 0.25 mm², p<0.001; Fig. 4D). In addition, VCAM1⁺FAP⁺ fibroblast density was positively correlated with both CXCL13⁺ cell density and CD20⁺ cell density (Spearman ρ = 0.362 and 0.766, respectively; Fig. 4E). TLS regions also showed significantly higher infiltration of VCAM1⁺FAP⁺ cells compared with non-TLS regions (369.5 [166.0-577.0] vs. 39.0 [5.0-134.0] cells per 0.25 mm², p<0.001; Fig. 4D). Furthermore, the abundance of VCAM1⁺FAP⁺ cells was positively correlated with the total TLS area per 0.25 mm² (Spearman ρ = 0.454, p<0.001; Fig. 4E).

To further assess the relevance of these signaling pathways in external cohorts, we examined the correlation between *CXCL13* and genes involved in the four selected ligand-receptor axes. In these RNA-seq cohorts, several genes within the VCAM, BTLA, and MHC-I signaling pathways positively correlated with *CXCL13* expression, whereas *SPP1*-related genes showed less consistent associations (Supplementary Fig. s6). These findings support a potential association between VCAM1⁺ fibroblast-rich stromal niches and CXCL13⁺ Tfh/B-cell-enriched immune organization in tumors of current smokers.

### Expression of *CXCL13* and selected TLS-associated genes is associated with overall survival in LUAD

**Figure 5:**
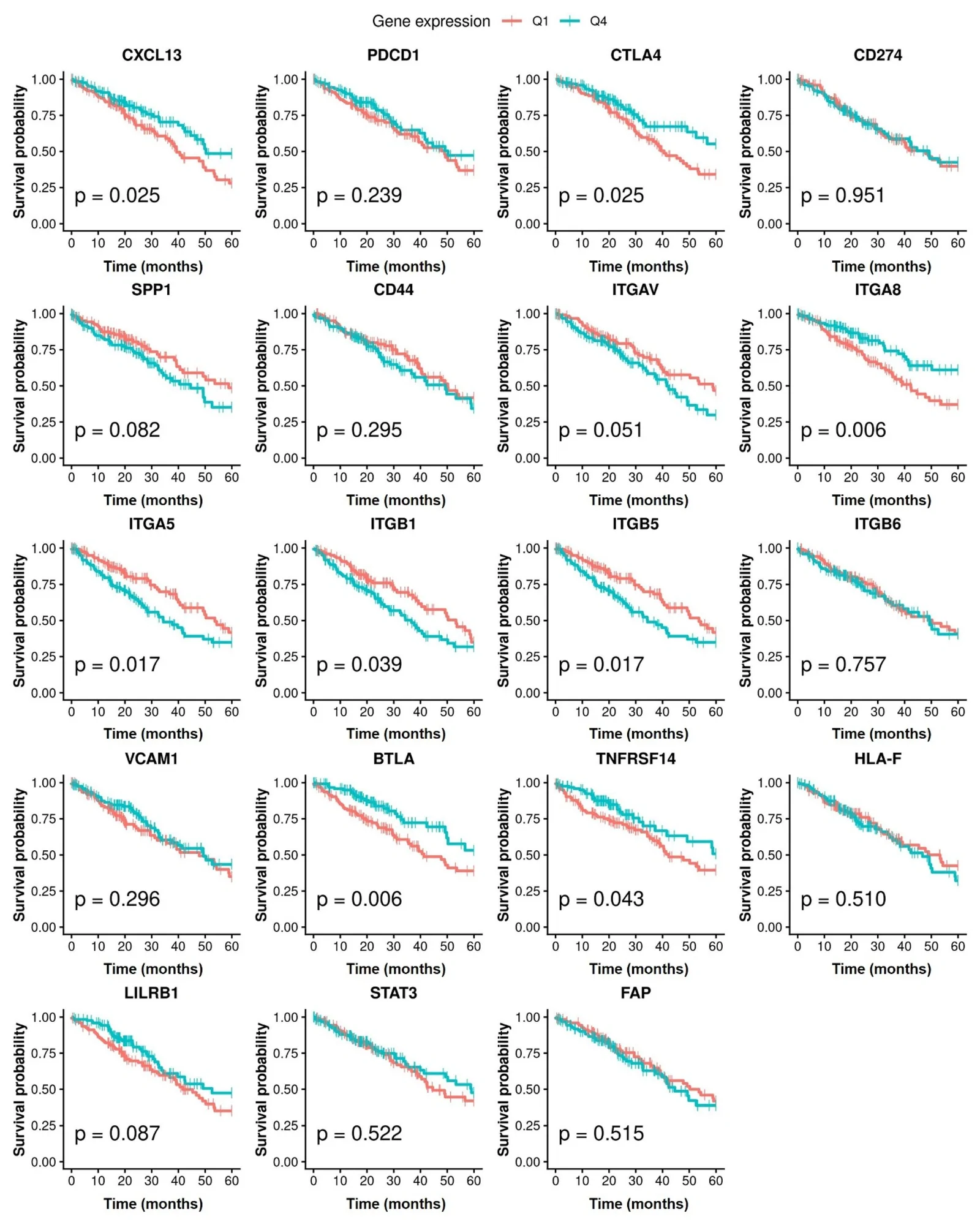
** Prognostic significance of CXCL13-associated signaling genes.** Kaplan-Meier survival curves comparing overall survival between patients with high (Q4) and low (Q1) expression of *CXCL13* and genes related to the SPP1, VCAM, BTLA, and MHC-I signaling pathways. Patients with higher expression of *CXCL13* and immune regulatory genes such as *CTLA4* and *BTLA* showed significantly improved survival, whereas several adhesion-related genes (e.g., integrins) also demonstrated prognostic associations. p-values were calculated using the log-rank test.

To explore the potential clinical relevance of the molecular profiles identified in our analyses, we evaluated their association with 5-year overall survival in an external large-scale LUAD bulk RNA-seq cohort from the TCGA GDC portal (Fig. 5). Candidate genes were selected from key molecules highlighted across the preceding Tfh-, immune checkpoint-, and ligand-receptor-related analyses among TLS-related cell clusters in the TME of smokers and never-smokers. Gene expression levels were z-score-normalized and patients were stratified into low- and high-expression groups (Q1 vs. Q4) for Kaplan-Meier survival analysis. High *CXCL13* expression was significantly associated with improved overall survival, supporting the potential prognostic relevance of the CXCL13⁺ Tfh-related immune program in LUAD. Among the selected candidate genes, higher expression of immune regulatory genes, including *CTLA4, BTLA*, and *TNFRSF14*, as well as adhesion-related genes, including *ITGA8, ITGA5, ITGB1*, and *ITGB5*, was associated with favorable survival outcomes. In contrast, several other genes, including *PDCD1, CD274, VCAM1*, and *FAP,* were not significantly associated with overall survival (Fig. 5). These findings suggest that the CXCL13-associated immune program, together with selected stromal interaction pathways, may be associated with favorable prognosis in LUAD.

## Discussion

In this study, we characterized the TME of early-stage LUAD according to smoking status, using integrated single-cell transcriptomic and spatial analyses. Current smoking was associated with enrichment of CXCL13⁺CD4⁺ T cells, increased B-cell accumulation, and enhanced TLS formation. However, these findings should not be interpreted as suggesting that smoking confers a favorable prognosis in NSCLC, nor should they support smoking behavior. It is important to distinguish between the intrinsic biological mechanisms identified in this study and any perceived beneficial effects of smoking.

In addition, cigarette smoking may remodel the LUAD TME in an exposure-duration-dependent manner 29. Ongoing or recent smoke exposure can rapidly induce oxidative stress, epithelial injury, and the release of pro-inflammatory cytokines, resulting in transient activation and recruitment of innate immune cells 30. In contrast, prolonged exposure may establish more persistent changes through cumulative genomic damage, epigenetic reprogramming, sustained adaptive immune dysregulation, and remodeling of fibroblasts and the extracellular matrix 31,32. Therefore, the TME observed in current smokers may reflect both acute inflammatory responses to recent exposure and chronic molecular and stromal alterations caused by long-term smoking. Several key findings were obtained in this study.

First, current smokers showed enrichment of CXCL13⁺ Tfh cells with relative depletion of CD4⁺ Trm cells, indicating smoking-associated remodeling of the CD4⁺ T-cell landscape toward a follicular helper-like phenotype. Previous studies have reported heterogeneous smoking-related immune alterations including T-cell dysfunction and inflammatory remodeling 33,34. Smoking is also linked to increased *CXCL13* expression and TLS formation in inflammatory lung diseases. Although direct evidence linking smoking to CXCL13⁺ Tfh cells and TLS formation in lung cancer remains limited, osteopontin, encoded by *SPP1*, has been implicated in Tfh differentiation and chronic inflammatory remodeling 35-37. In our study, CellChat analysis also predicted relatively greater SPP1 signaling activity in tumors from smokers, consistent with previous reports showing smoking-associated *SPP1* upregulation in lung tissues. Together with previous reports, these computational observations raise the possibility that smoking-associated inflammatory niches may contribute to CXCL13⁺ Tfh enrichment and TLS organization.

Second, CXCL13⁺ Tfh cells highly expressed immune checkpoint-related molecules, including *PDCD1* and *CTLA4*. In external validation datasets, *CXCL13* expression was also positively associated with checkpoint-related programs. This checkpoint-enriched phenotype is consistent with previous studies of CXCL13-producing Tfh-like cells 38,39. In addition, recent studies have associated CXCL13⁺ cells and TLS-rich TMEs with improved responses to ICIs across several cancers 40-42. Consistent with these findings, higher *CXCL13* expression was associated with favorable survival outcomes in this study. These observations may partially explain the improved responsiveness to ICIs observed in smokers, although further prospective validation is required.

Third, CXCL13⁺ Tfh cells and VCAM1⁺ fibroblasts were spatially associated with TLS-related regions. In parallel, CellChat analysis predicted relatively greater VCAM-related signaling involving fibroblasts in the tumors from current smokers. Together, these observations suggest an association between VCAM1⁺ fibroblasts and TLS-related stromal organization, but do not establish direct cellular crosstalk or causality.

Previous studies have highlighted the importance of CXCL13⁺ T cells and fibroblasts in TLS development across multiple cancer types 43-48. Although our findings are associative, they support the potential role of VCAM1⁺ fibroblasts in smoking-associated TLS formation. However, neither bulk *VCAM1* nor *FAP* expression was significantly associated with overall survival in the TCGA LUAD cohort. This finding indicates that the spatial association of VCAM1⁺FAP⁺ fibroblasts with TLS-related immune regions should not be interpreted as evidence of a protective or favorable prognostic function. Cancer-associated fibroblasts (CAFs) are functionally heterogeneous, and *VCAM1* expression may be shared by fibroblast states with distinct or even opposing effects on tumor immunity and progression. Furthermore, bulk RNA-sequencing cannot distinguish *VCAM1* expression in VCAM1⁺FAP⁺ fibroblasts from that in other stromal, endothelial, or immune cell populations, nor can it estimate the abundance of this specific fibroblast subset. Therefore, the functional and prognostic significance of VCAM1⁺FAP⁺ fibroblasts remains uncertain and requires cell-type-specific and functional validation.

This study has several limitations. First, our conclusions regarding cellular interactions were primarily based on associations observed through immunofluorescence staining, rather than direct functional validation. Although we complemented these findings with single-cell transcriptomic analyses and external validation using publicly available datasets, we were unable to perform *in vitro* or *in vivo* functional experiments such as cell-based assays or animal models. Therefore, additional mechanistic studies are required to establish causal relationships. An additional limitation is that smoking exposure cannot be experimentally controlled in human subjects for ethical reasons. Accordingly, this study was necessarily observational in design, and residual confounding cannot be fully excluded. To mitigate this limitation, we compared clearly defined current smokers and never-smokers, used paired tumor and adjacent normal tissues, and incorporated orthogonal validation across independent public scRNA-seq datasets, an external immunofluorescence cohort, and external bulk RNA-sequencing cohorts. Second, multiplex immunofluorescence was conducted using a four-channel platform, which limited our ability to directly interrogate complex cell-cell interactions at higher dimensional resolution. To address this limitation, we performed high-resolution whole-slide scanning and detailed spatial quantification, which enabled comprehensive assessment of cell distribution and TLS-related features. Nevertheless, future studies using higher-plex imaging technologies may provide a more precise characterization of cellular interactions within the TLS. Third, the discovery dataset was derived from a single center and included only ten patients, which limited statistical power and increased susceptibility to patient-specific or outlier effects. Although no statistically significant differences were observed in age, TNM stage, or *EGFR* mutation status between current smokers and never-smokers, the small sample size precludes firm conclusions regarding baseline comparability. In addition, sex distribution was significantly imbalanced: all current smokers were male, whereas four of the five never-smokers were female. This pattern partly reflects the strong association between smoking status and sex in the Korean population; however, sex may independently influence the tumor immune microenvironment and therefore represents an important potential confounding factor. Accordingly, the observed differences should be regarded as exploratory and require confirmation in larger, sex-balanced, multicenter cohorts.

Our findings should not be interpreted as supporting smoking behavior. Understanding the intrinsic mechanisms underlying smoking-associated immune remodeling may provide insights into tumor immune organization and inform future immunotherapeutic strategies. Emerging evidence suggests that CXCL13 has clinical potential not only as a biomarker for favorable prognosis, but also as an adjunctive immunotherapeutic target 49,50. By contrast, direct therapeutic evidence for VCAM-1 remains limited; however, given its role in leukocyte recruitment and retention, fibroblast-associated VCAM-1 may represent a stromal program that cooperates with CXCL13-driven immune organization and could be explored in future combinatorial strategies 51-53.

## Conclusions

In this study, CXCL13⁺ Tfh cells were enriched in the TME of current smokers with LUAD and exhibited increased expression of ICI-related genes. CXCL13⁺ Tfh cells and VCAM1⁺FAP⁺ fibroblasts were enriched in tumors from current smokers and spatially associated with TLS-rich regions. These findings suggest that they are components of smoking-associated TLS-related immune organization, although their functional interactions and clinical implications remain to be established. These findings may provide biological relevance, rather than direct evidence, for the differential ICI responsiveness previously reported according to smoking history and support further evaluation of immune and stromal features as candidate biomarkers in ICI-treated cohorts. Further mechanistic and prospective clinical studies are needed to validate these findings.

## Supplementary Material

Supplementary figures and tables.

## Data Availability

The datasets are available in the NCBI Sequence Read Archive under BioProject accession numbers PRJNA773987 and PRJNA901260.

## Abbreviations

CAF: Cancer-associated fibroblast
ICI: immune checkpoint inhibitor
LUAD: lung adenocarcinoma
NSCLC: non-small cell lung cancer
TME: tumor microenvironment
TLS: tertiary lymphoid structure
