# Supplementary material for: Smoking-Associated Enrichment of CXCL13+ Tfh Cells and VCAM1+ Fibroblasts Is Linked to Tertiary Lymphoid Structure Formation in Lung Adenocarcinoma
Source: J Cancer. 2026 Sep 3;17(9):1626–38. doi: 10.7150/jca.138884 (PMC13573297; doi:10.7150/jca.138884)
Supplement: Supplementary file 1 [file jcav17p1626s1.pdf]

Supplementary materials

Table of contents

Supplementary Tables

**Appendix Table 1**	Baseline characteristics
**Appendix Table 2**	Comparison of baseline clinical characteristics between current smokers and never-smokers
**Appendix Table 3**	Result of quality control for raw sequencing data
**Appendix Table 4**	Proportion of major cell lineage
**Appendix Table 5**	Proportion of T/NK-cell subtypes according to smoking status

Supplementary Figures

**Supplementary Figure 1**	Single-cell landscape of lung tissue across discovery and validation cohorts
**Supplementary Figure 2**	Marker gene expression and gene ontology analysis
**Supplementary Figure 3**	Cell enrichment analysis of T and NK subsets from tumor tissue
**Supplementary Figure 4**	CXCL13 expression is associated with immune checkpoint activation across cohorts and tissue validation
**Supplementary Figure 5**	Smoking-associated alterations in CellChat-predicted cell–cell communication patterns in tumor and normal tissues.
**Supplementary Figure 6**	External validation of CXCL13-associated signaling gene expression across independent cohorts

Appendix Table 1. Baseline characteristics

Sample ID	Original patient ID	Age	Sex	Smoking history	Sample origin	Stage	EGFR
Sample001	Case_007	66	M	Current smokers	Tumor	IA	EGFR-WT
Sample002	Case_007	66	M	Current smokers	Normal	IA	EGFR-WT
Sample003	Case_008	61	M	Current smokers	Tumor	IA	EGFR-WT
Sample004	Case_008	61	M	Current smokers	Normal	IA	EGFR-WT
Sample005	Case_009	66	M	Current smokers	Tumor	IA	EGFR-WT
Sample006	Case_009	66	M	Current smokers	Normal	IA	EGFR-WT
Sample007	Case_010	66	M	Current smokers	Tumor	IA	EGFR-MT
Sample008	Case_010	66	M	Current smokers	Normal	IA	EGFR-MT
Sample009	Case_012	60	M	Current smokers	Tumor	IIB	EGFR-WT
Sample010	Case_012	60	M	Current smokers	Normal	IIB	EGFR-WT
Sample011	Case_001	60	F	Never-smokers	Tumor	IB	EGFR-MT
Sample012	Case_001	60	F	Never-smokers	Normal	IB	EGFR-MT
Sample013	Case_002	76	F	Never-smokers	Tumor	IA	EGFR-MT
Sample014	Case_002	76	F	Never-smokers	Normal	IA	EGFR-MT
Sample015	Case_003	71	F	Never-smokers	Tumor	IA	EGFR-MT
Sample016	Case_003	71	F	Never-smokers	Normal	IA	EGFR-MT
Sample017	Case_004	73	M	Never-smokers	Tumor	IA	EGFR-WT
Sample018	Case_004	73	M	Never-smokers	Normal	IA	EGFR-WT
Sample019	Case_005	68	F	Never-smokers	Tumor	IA	EGFR-WT
Sample020	Case_005	68	F	Never-smokers	Normal	IA	EGFR-WT
Validation001	SSN24	67	M	Current smokers	Tumor	IB	Unknown
Validation002	SSN30	67	F	Never-smokers	Tumor	IB	Unknown
Validation003	LUNG_T08	Unknown	Unknown	Never-smokers	Tumor	IB	EGFR-MT
Validation004	LUNG_N08	Unknown	Unknown	Never-smokers	Normal	IB	EGFR-MT
Validation005	LUNG_T19	Unknown	Unknown	Current smokers	Tumor	IA	EGFR-MT
Validation006	LUNG_N19	Unknown	Unknown	Current smokers	Normal	IA	EGFR-MT
Validation007	LUNG_T30	Unknown	Unknown	Never-smokers	Tumor	IA	EGFR-MT
Validation008	LUNG_N30	Unknown	Unknown	Never-smokers	Normal	IA	EGFR-MT
Validation009	LUNG_T20	Unknown	Unknown	Current smokers	Tumor	IA	EGFR-WT
Validation010	LUNG_N20	Unknown	Unknown	Current smokers	Normal	IA	EGFR-WT
Validation011	LUNG_T34	Unknown	Unknown	Never-smokers	Tumor	IA	EGFR-WT

Validation012	LUNG_N34	Unknown	Unknown	Never-smokers	Normal	IA	EGFR-WT

Note: Each discovery-cohort patient consists of paired tumor and adjacent normal samples; therefore, patient characteristics are repeated for the two corresponding samples.

Abbreviations: EGFR, epidermal growth factor receptor; MT, mutant; WT, wild type.

Appendix Table 2. Comparison of baseline clinical characteristics between current smokers and never-smokers

Characteristics	Current smokers (N=5)	Never-smokers (N=5)	p-value
Age, years	66 [61–66]	71 [68–73]	0.112
Sex			0.048
Male	5 (100.0%)	1 (20.0%)	
Female	0 (0.0%)	4 (80.0%)	
Pathologic stage			1.000
IA	4 (80.0%)	4 (80.0%)	
IB	0 (0.0%)	1 (20.0%)	
IIB	1 (20.0%)	0 (0.0%)	
EGFR mutation status			0.524
EGFR-WT	4 (80.0%)	2 (40.0%)	
EGFR-MT	1 (20.0%)	3 (60.0%)	

Data are presented as median (interquartile range) or number (percentage). Continuous variables were compared using the Mann–Whitney U test, and categorical variables were compared using Fisher's exact test. The overall distribution of TNM stage was compared using the Fisher–Freeman–Halton exact test. Because of the small sample size, P values should be interpreted with caution.

Abbreviations: EGFR, epidermal growth factor receptor; IQR, interquartile range; TNM, tumor–node–metastasis.

Appendix Table 3. Results of quality control for raw sequencing data

Sample ID	Raw	After detection-based QC		After mitochondrial- and ribosomal-read-based QC		DoubletFinder	Final
	Cells	Cells	RNA	Cells	RNA	Doublet count	Cell count
Sample001	9475	9413	24229	5176	24215	206	4970
Sample002	13135	12728	23792	10054	23778	772	9282
Sample003	14957	14818	26654	12473	26640	1187	11286
Sample004	19441	19423	24114	17493	24100	2331	15162
Sample005	3798	3744	25437	2961	25423	68	2893
Sample006	8709	8688	24908	7422	24894	422	7000
Sample007	9295	9279	25220	5395	25206	224	5171
Sample008	3505	3421	23558	2515	23544	49	2466
Sample009	8262	8115	24023	5790	24009	257	5533
Sample010	11878	11808	22817	8315	22803	529	7786
Sample011	8820	8386	23770	2684	23756	56	2628
Sample012	11616	11429	22978	8098	22964	502	7596
Sample013	10563	10549	22591	2629	22577	54	2575
Sample014	11363	10775	23218	7781	23204	464	7317
Sample015	9790	9787	25631	6675	25617	342	6333
Sample016	10461	9921	25754	8485	25740	551	7934
Sample017	6642	6568	23994	5468	23980	230	5238
Sample018	13261	12919	25194	10910	25180	909	10001
Sample019	6869	6869	25438	4917	25424	186	4731
Sample020	13834	13726	25384	11752	25370	1054	10698
Validation001	7525	7389	21095	6252	21081	300	5952
Validation002	8832	8160	20001	7017	19987	377	6640
Validation003	4094	4004	19060	3728	19046	107	3621
Validation004	3832	3736	19327	3317	19313	85	3232
Validation005	4626	4548	19855	4259	19841	140	4119
Validation006	4276	4234	19016	3917	19002	119	3798
Validation007	4632	4590	19413	4431	19399	151	4280
Validation008	4123	4068	19264	3816	19250	113	3703
Validation009	4522	4470	19069	4035	19055	126	3909
Validation010	6401	6274	19193	5691	19179	249	5442
Validation011	4067	4008	20295	2794	20281	61	2733
Validation012	5887	5755	20063	5224	20049	210	5014

Abbreviations: QC, quality control; RNA, ribonucleic acid.

Appendix Table 4. Proportions of major cell lineages

History of smoking	Never-smoker			Current smoker		
**Tissue type**	**Normal**	**Tumor**	**p-value**	**Normal**	**Tumor**	**p-value**
**Discovery dataset**	(N=43546)	(N=21505)	<0.001	(N=41696)	(N=29853)	<0.001
- T/NK cells	22336 (51.3%)	6435 (29.9%)		6826 (16.4%)	9941 (33.3%)	
- B cells	139 (0.3%)	379 (1.8%)		227 (0.5%)	1872 (6.3%)	
- Myeloid cells	8453 (19.4%)	4321 (20.1%)		27471 (65.9%)	6960 (23.3%)	
- Epithelial cells	9447 (21.7%)	7770 (36.1%)		3420 (8.2%)	5827 (19.5%)	
- Endothelial cells	1520 (3.5%)	764 (3.6%)		834 (2.0%)	723 (2.4%)	
- Fibroblasts	611 (1.4%)	694 (3.2%)		1516 (3.6%)	1929 (6.5%)	
- Mast cells	886 (2.0%)	909 (4.2%)		1044 (2.5%)	768 (2.6%)	
- Proliferative cells	154 (0.4%)	233 (1.1%)		358 (0.9%)	1833 (6.1%)	
**Validation dataset**	(N=11949)	(N=17274)	<0.001	(N=9240)	(N=13980)	<0.001
- T/NK cells	5825 (48.7%)	7657 (44.3%)		3567 (38.6%)	6257 (44.8%)	
- B cells	106 (0.9%)	2778 (16.1%)		45 (0.5%)	643 (4.6%)	
- Myeloid cells	3601 (30.1%)	1607 (9.3%)		4245 (45.9%)	2907 (20.8%)	
- Epithelial cells	1157 (9.7%)	3797 (22.0%)		312 (3.4%)	2520 (18.0%)	
- Endothelial cells	505 (4.2%)	239 (1.4%)		564 (6.1%)	513 (3.7%)	
- Fibroblasts	493 (4.1%)	335 (1.9%)		115 (1.2%)	307 (2.2%)	
- Mast cells	203 (1.7%)	804 (4.7%)		354 (3.8%)	618 (4.4%)	
- Proliferative cells	59 (0.5%)	57 (0.3%)		38 (0.4%)	215 (1.5%)	

Data are presented as number of cells (percentage of the total cells in each tissue group). P values represent comparisons of the overall cell-type distributions between normal and tumor tissues within each smoking group and were calculated using the chi-square test.

Abbreviations: NK, natural killer; N, number of cells.

Appendix Table 5. Proportion of T/NK-cell subtypes according to smoking status

Dataset	Tissue	T/NK-cell subtype	Never-smoker n (%)	Current smoker n (%)	p-value	BH-adjusted p-value
Discovery	Non-tumor	Naïve	1,432 (6.6%)	442 (6.8%)	0.551	0.551
Discovery	Non-tumor	CD4-Eff1	776 (3.6%)	478 (7.4%)	<0.001	<0.001
Discovery	Non-tumor	CD4-Eff2	547 (2.5%)	314 (4.9%)	<0.001	<0.001
Discovery	Non-tumor	CD4-Treg	197 (0.9%)	109 (1.7%)	<0.001	<0.001
Discovery	Non-tumor	CD4-Trm	1,682 (7.8%)	364 (5.6%)	<0.001	<0.001
Discovery	Non-tumor	CD4-Tfh	22 (0.1%)	14 (0.2%)	0.029	0.033
Discovery	Non-tumor	CD8-Trm1	611 (2.8%)	270 (4.2%)	<0.001	<0.001
Discovery	Non-tumor	CD8-Trm2	246 (1.1%)	324 (5.0%)	<0.001	<0.001
Discovery	Non-tumor	CD8-Tem	1,686 (7.8%)	622 (9.6%)	<0.001	<0.001
Discovery	Non-tumor	CD8-Tex	96 (0.4%)	45 (0.7%)	0.016	0.019
Discovery	Non-tumor	CD8-Eff1	2,606 (12.1%)	396 (6.1%)	<0.001	<0.001
Discovery	Non-tumor	CD8-Eff2	2,188 (10.1%)	866 (13.4%)	<0.001	<0.001
Discovery	Non-tumor	NK-Low	4,067 (18.9%)	356 (5.5%)	<0.001	<0.001
Discovery	Non-tumor	NK-Act	2,046 (9.5%)	766 (11.9%)	<0.001	<0.001
Discovery	Non-tumor	NK-Eff	2,377 (11.0%)	735 (11.4%)	0.416	0.442
Discovery	Non-tumor	NK-Inhib	545 (2.5%)	261 (4.0%)	<0.001	<0.001
Discovery	Non-tumor	NK-Rest	447 (2.1%)	93 (1.4%)	<0.001	0.001
Discovery	Tumor	Naïve	1,034 (17.3%)	1,119 (12.0%)	<0.001	<0.001
Discovery	Tumor	CD4-Eff1	397 (6.6%)	723 (7.8%)	0.009	0.012
Discovery	Tumor	CD4-Eff2	251 (4.2%)	418 (4.5%)	0.395	0.395
Discovery	Tumor	CD4-Treg	768 (12.8%)	1,642 (17.6%)	<0.001	<0.001
Discovery	Tumor	CD4-Trm	870 (14.5%)	708 (7.6%)	<0.001	<0.001
Discovery	Tumor	CD4-Tfh	155 (2.6%)	622 (6.7%)	<0.001	<0.001
Discovery	Tumor	CD8-Trm1	397 (6.6%)	538 (5.8%)	0.032	0.039
Discovery	Tumor	CD8-Trm2	152 (2.5%)	370 (4.0%)	<0.001	<0.001
Discovery	Tumor	CD8-Tem	813 (13.6%)	1,039 (11.2%)	<0.001	<0.001
Discovery	Tumor	CD8-Tex	215 (3.6%)	1,103 (11.8%)	<0.001	<0.001
Discovery	Tumor	CD8-Eff1	156 (2.6%)	183 (2.0%)	0.010	0.012
Discovery	Tumor	CD8-Eff2	140 (2.3%)	241 (2.6%)	0.366	0.389
Discovery	Tumor	NK-Low	240 (4.0%)	125 (1.3%)	<0.001	<0.001
Discovery	Tumor	NK-Act	96 (1.6%)	88 (0.9%)	<0.001	<0.001
Discovery	Tumor	NK-Eff	107 (1.8%)	61 (0.7%)	<0.001	<0.001
Discovery	Tumor	NK-Inhib	40 (0.7%)	144 (1.5%)	<0.001	<0.001
Discovery	Tumor	NK-Rest	153 (2.6%)	194 (2.1%)	0.058	0.066
Validation	Non-tumor	Naïve	265 (5.1%)	100 (3.0%)	<0.001	<0.001
Validation	Non-tumor	CD4-Eff1	61 (1.2%)	45 (1.3%)	0.485	0.549
Validation	Non-tumor	CD4-Eff2	482 (9.3%)	528 (15.8%)	<0.001	<0.001
Validation	Non-tumor	CD4-Treg	60 (1.2%)	28 (0.8%)	0.187	0.228
Validation	Non-tumor	CD4-Trm	374 (7.2%)	60 (1.8%)	<0.001	<0.001
Validation	Non-tumor	CD4-Tfh	1 (0.0%)	1 (0.0%)	1.000	1.000
Validation	Non-tumor	CD8-Trm1	18 (0.3%)	50 (1.5%)	<0.001	<0.001
Validation	Non-tumor	CD8-Trm2	48 (0.9%)	267 (8.0%)	<0.001	<0.001
Validation	Non-tumor	CD8-Tem	491 (9.4%)	129 (3.9%)	<0.001	<0.001
Validation	Non-tumor	CD8-Tex	10 (0.2%)	12 (0.4%)	0.188	0.228
Validation	Non-tumor	CD8-Eff1	316 (6.1%)	27 (0.8%)	<0.001	<0.001
Validation	Non-tumor	CD8-Eff2	451 (8.7%)	262 (7.8%)	0.186	0.228
Validation	Non-tumor	NK-Low	1,137 (21.8%)	260 (7.8%)	<0.001	<0.001
Validation	Non-tumor	NK-Act	26 (0.5%)	10 (0.3%)	0.175	0.228
Validation	Non-tumor	NK-Eff	1,364 (26.2%)	1,517 (45.4%)	<0.001	<0.001
Validation	Non-tumor	NK-Inhib	28 (0.5%)	20 (0.6%)	0.767	0.815
Validation	Non-tumor	NK-Rest	73 (1.4%)	26 (0.8%)	0.009	0.016
Validation	Tumor	Naïve	2,388 (35.7%)	1,079 (18.5%)	<0.001	<0.001
Validation	Tumor	CD4-Eff1	221 (3.3%)	82 (1.4%)	<0.001	<0.001
Validation	Tumor	CD4-Eff2	1,078 (16.1%)	1,195 (20.5%)	<0.001	<0.001

Validation	Tumor	CD4-Treg	537 (8.0%)	419 (7.2%)	0.079	0.096
Validation	Tumor	CD4-Trm	462 (6.9%)	189 (3.2%)	<0.001	<0.001
Validation	Tumor	CD4-Tfh	122 (1.8%)	389 (6.7%)	<0.001	<0.001
Validation	Tumor	CD8-Trm1	64 (1.0%)	242 (4.1%)	<0.001	<0.001
Validation	Tumor	CD8-Trm2	166 (2.5%)	422 (7.2%)	<0.001	<0.001
Validation	Tumor	CD8-Tem	902 (13.5%)	752 (12.9%)	0.341	0.341
Validation	Tumor	CD8-Tex	55 (0.8%)	275 (4.7%)	<0.001	<0.001
Validation	Tumor	CD8-Eff1	86 (1.3%)	61 (1.0%)	0.244	0.260
Validation	Tumor	CD8-Eff2	135 (2.0%)	281 (4.8%)	<0.001	<0.001
Validation	Tumor	NK-Low	233 (3.5%)	133 (2.3%)	<0.001	<0.001
Validation	Tumor	NK-Act	25 (0.4%)	9 (0.2%)	0.024	0.031
Validation	Tumor	NK-Eff	32 (0.5%)	204 (3.5%)	<0.001	<0.001
Validation	Tumor	NK-Inhib	54 (0.8%)	10 (0.2%)	<0.001	<0.001
Validation	Tumor	NK-Rest	133 (2.0%)	93 (1.6%)	0.106	0.121

Data are presented as number of cells (percentage of the total retained T/NK cells in each tissue and smoking group). Putative contaminating clusters with alveolar-cell or B-cell signatures were excluded before analysis. p-values represent comparisons of the proportion of each T/NK-cell subtype between never-smokers and current smokers within tumor or non-tumor tissues and were calculated using Fisher's exact test by comparing each subtype with all remaining T/NK-cell subtypes. Benjamini–Hochberg-adjusted p-values were calculated separately within tumor and non-tumor tissues.

Abbreviations: Act, activated; Eff, effector; NK, natural killer; N, number of cells; Tem, effector memory T cell; Tex, exhausted T cell; Tfh, T follicular helper cell; Treg, regulatory T cell; Trm, tissue-resident memory T cell.

Supplementary Figure 1. Single-cell landscape of lung tissue across discovery and validation cohorts

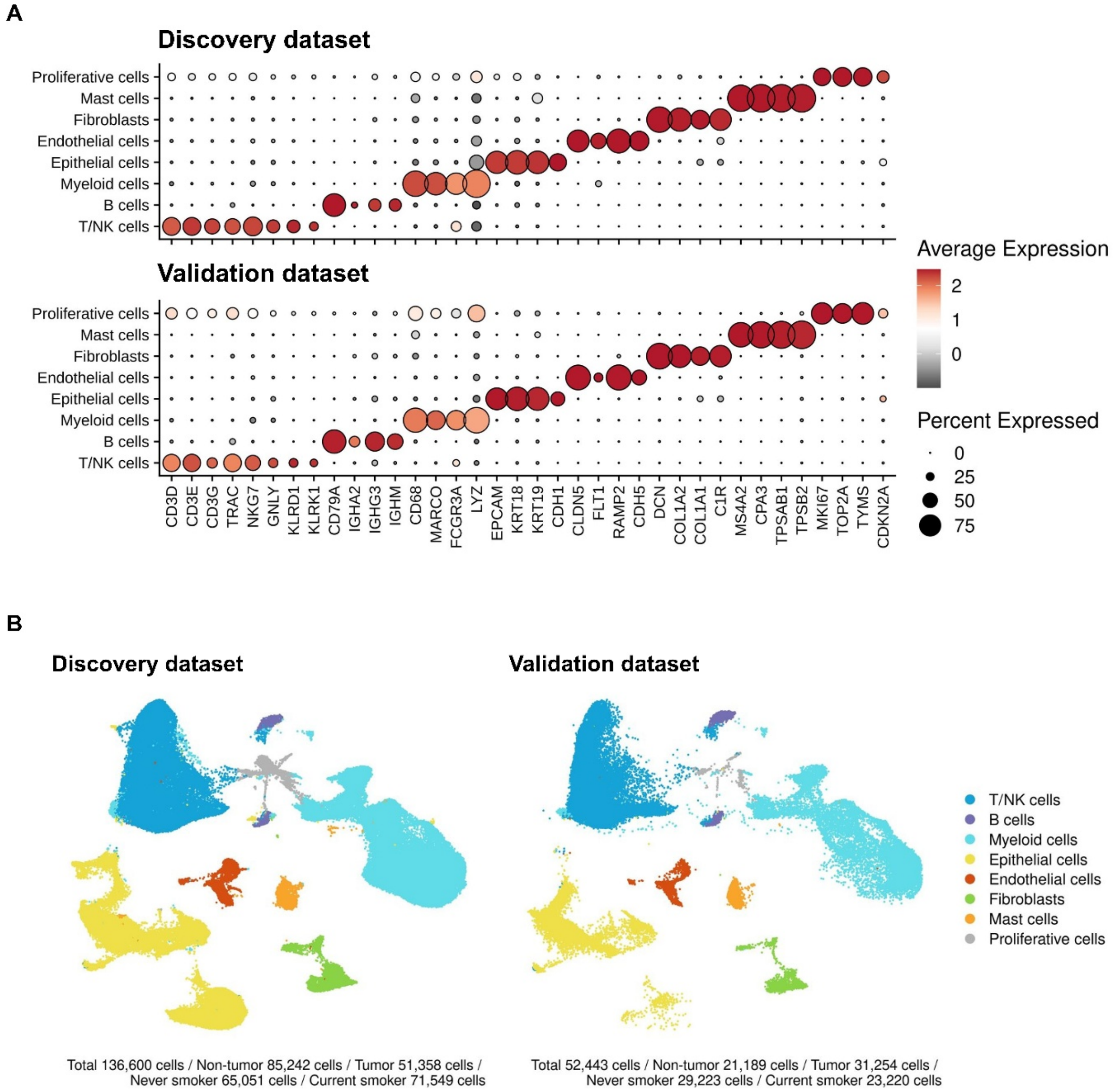

C

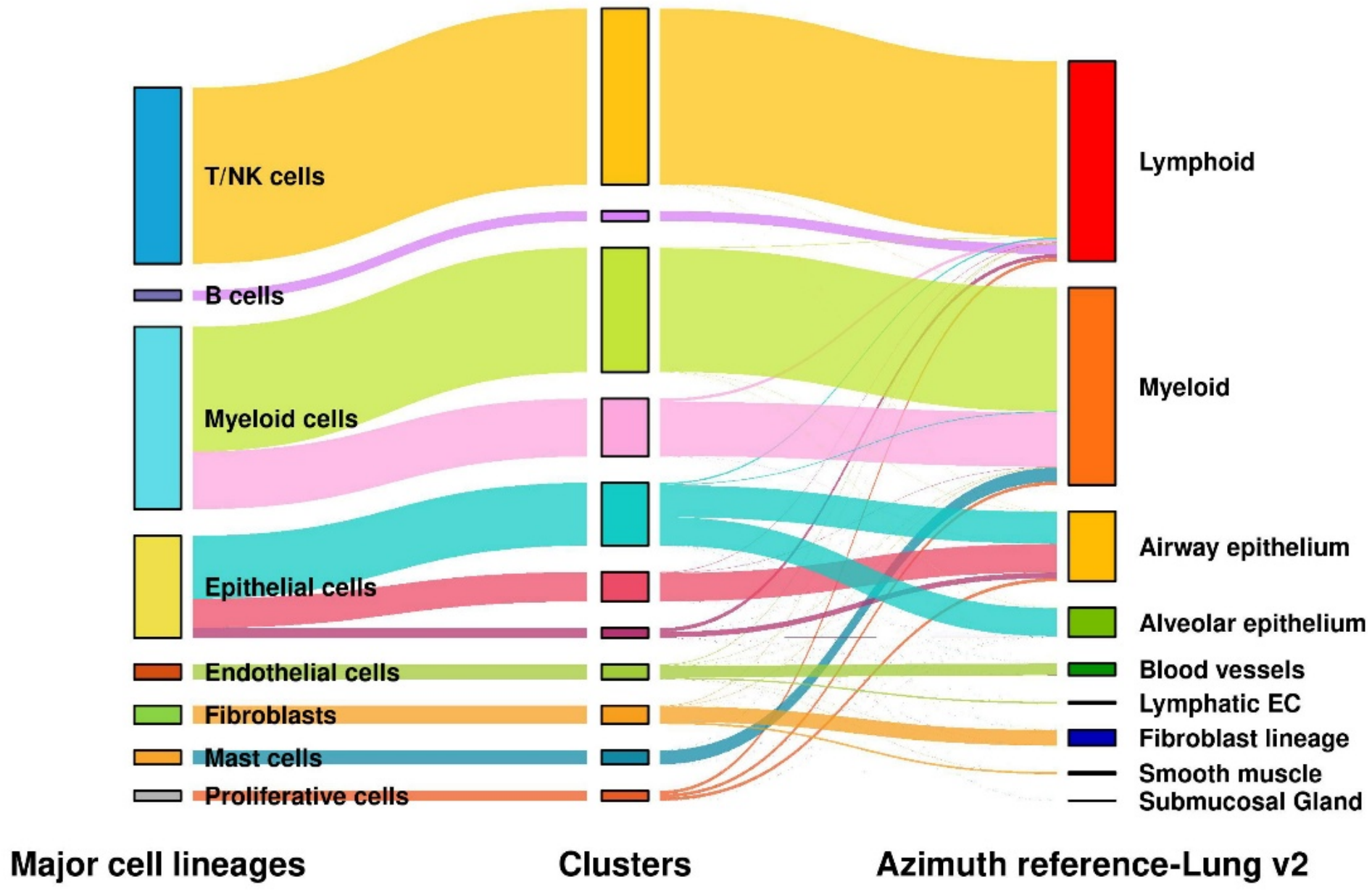

(A) Canonical marker gene expression across major cell lineages

(B) UMAP visualization of discovery and validation cohorts

(C) Cell lineage annotation mapped to Azimuth lung reference (v2)

Abbreviations: NK, natural killer

Supplementary Figure 2. Marker gene expression and gene ontology analysis

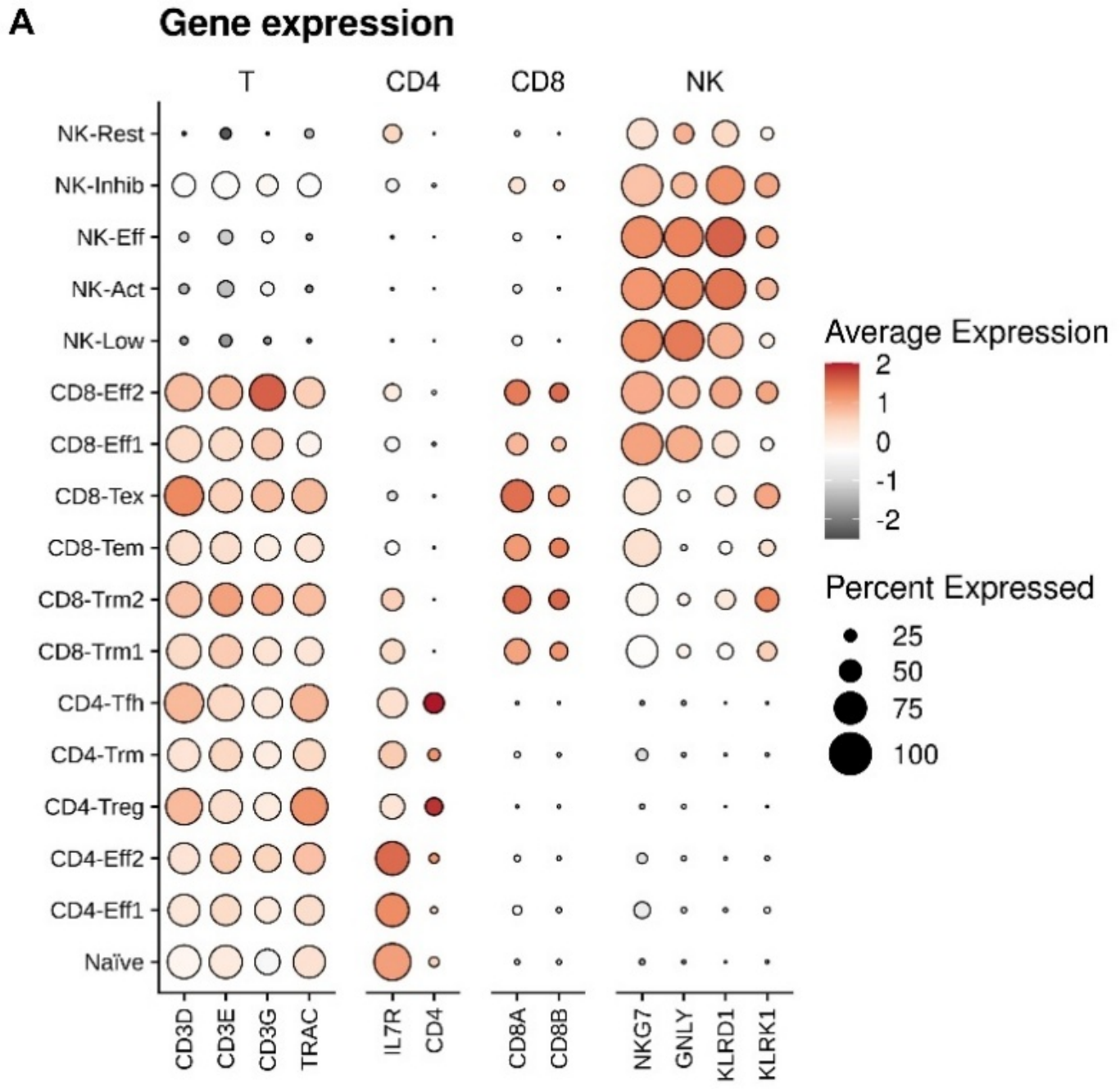

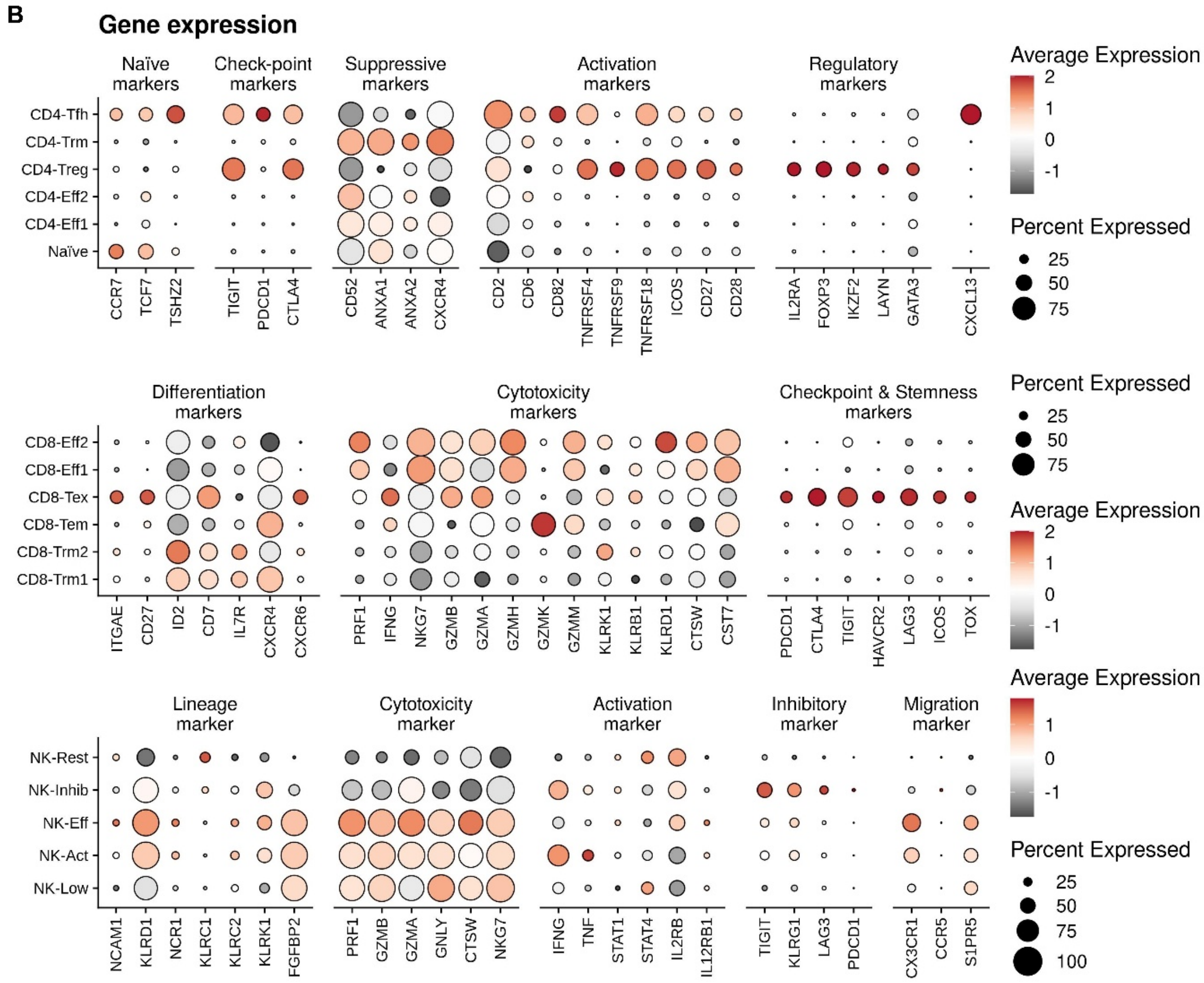

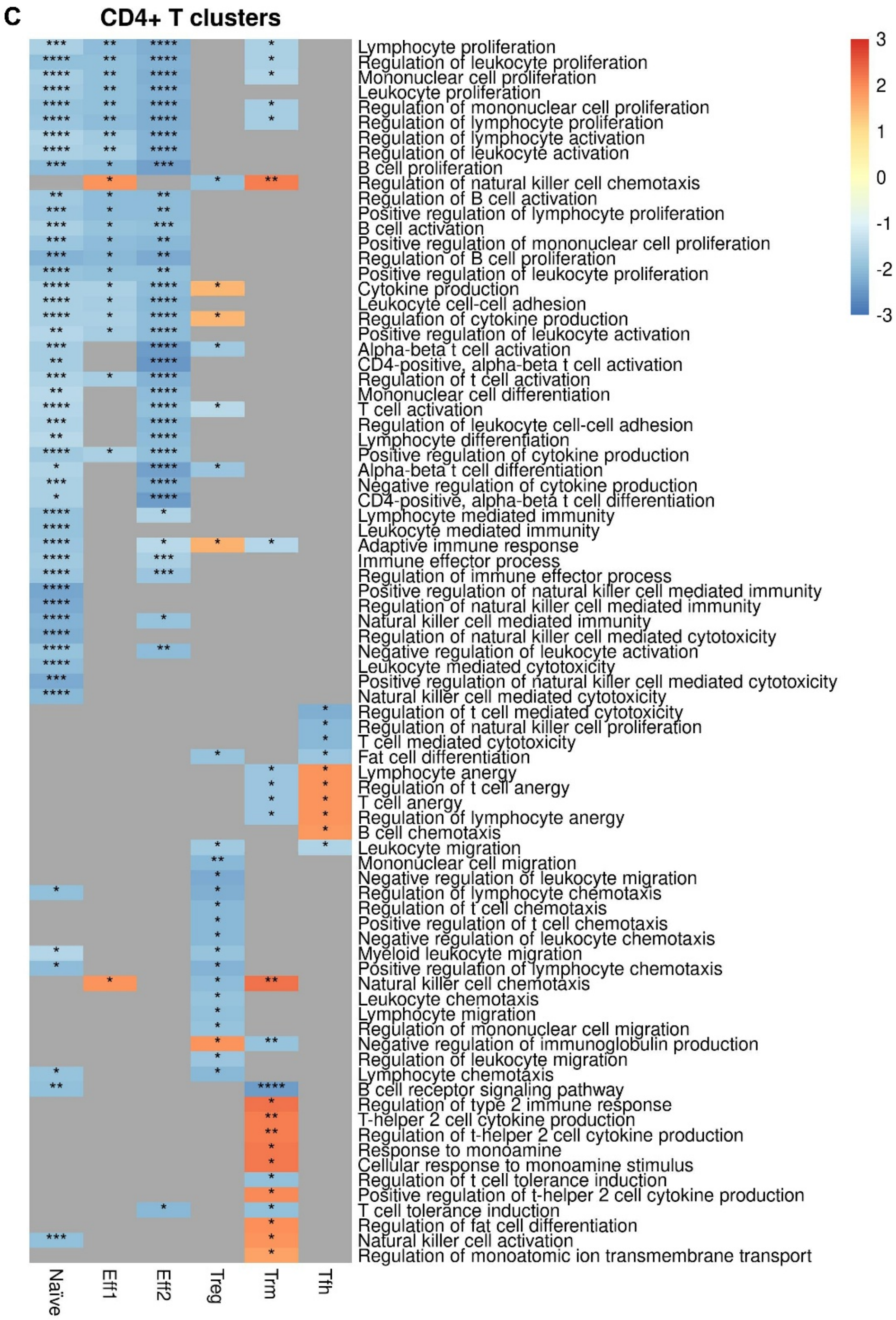
C
CD4+ T clusters
Lymphocyte proliferation
Regulation of leukocyte proliferation
Mononuclear cell proliferation
Leukocyte proliferation
Regulation of mononuclear cell proliferation
Regulation of lymphocyte proliferation
Regulation of lymphocyte activation
Regulation of leukocyte activation
B cell proliferation
Regulation of natural killer cell chemotaxis
Regulation of B cell activation
Positive regulation of lymphocyte proliferation
B cell activation
Positive regulation of mononuclear cell proliferation
Regulation of B cell proliferation
Positive regulation of leukocyte proliferation
Cytokine production
Leukocyte cell-cell adhesion
Regulation of cytokine production
Positive regulation of leukocyte activation
Alpha-beta t cell activation
CD4-positive, alpha-beta t cell activation
Regulation of t cell activation
Mononuclear cell differentiation
T cell activation
Regulation of leukocyte cell-cell adhesion
Lymphocyte differentiation
Positive regulation of cytokine production
Alpha-beta t cell differentiation
Negative regulation of cytokine production
CD4-positive, alpha-beta t cell differentiation
Lymphocyte mediated immunity
Leukocyte mediated immunity
Adaptive immune response
Immune effector process
Regulation of immune effector process
Positive regulation of natural killer cell mediated immunity
Regulation of natural killer cell mediated immunity
Natural killer cell mediated immunity
Regulation of natural killer cell mediated cytotoxicity
Negative regulation of leukocyte activation
Leukocyte mediated cytotoxicity
Positive regulation of natural killer cell mediated cytotoxicity
Natural killer cell mediated cytotoxicity
Regulation of t cell mediated cytotoxicity
Regulation of natural killer cell proliferation
T cell mediated cytotoxicity
Fat cell differentiation
Lymphocyte anergy
Regulation of t cell anergy
T cell anergy
Regulation of lymphocyte anergy
B cell chemotaxis
Leukocyte migration
Mononuclear cell migration
Negative regulation of leukocyte migration
Regulation of lymphocyte chemotaxis
Regulation of t cell chemotaxis
Positive regulation of t cell chemotaxis
Negative regulation of leukocyte chemotaxis
Myeloid leukocyte migration
Positive regulation of lymphocyte chemotaxis
Natural killer cell chemotaxis
Leukocyte chemotaxis
Lymphocyte migration
Regulation of mononuclear cell migration
Negative regulation of immunoglobulin production
Regulation of leukocyte migration
Lymphocyte chemotaxis
B cell receptor signaling pathway
Regulation of type 2 immune response
T-helper 2 cell cytokine production
Regulation of t-helper 2 cell cytokine production
Response to monoamine
Cellular response to monoamine stimulus
Regulation of t cell tolerance induction
Positive regulation of t-helper 2 cell cytokine production
T cell tolerance induction
Regulation of fat cell differentiation
Natural killer cell activation
Regulation of monoatomic ion transmembrane transport
Naïve
Eff1
Eff2
Treg
Trm
Tfh
3
2
1
0
-1
-2
-3

D CD8+ T clusters

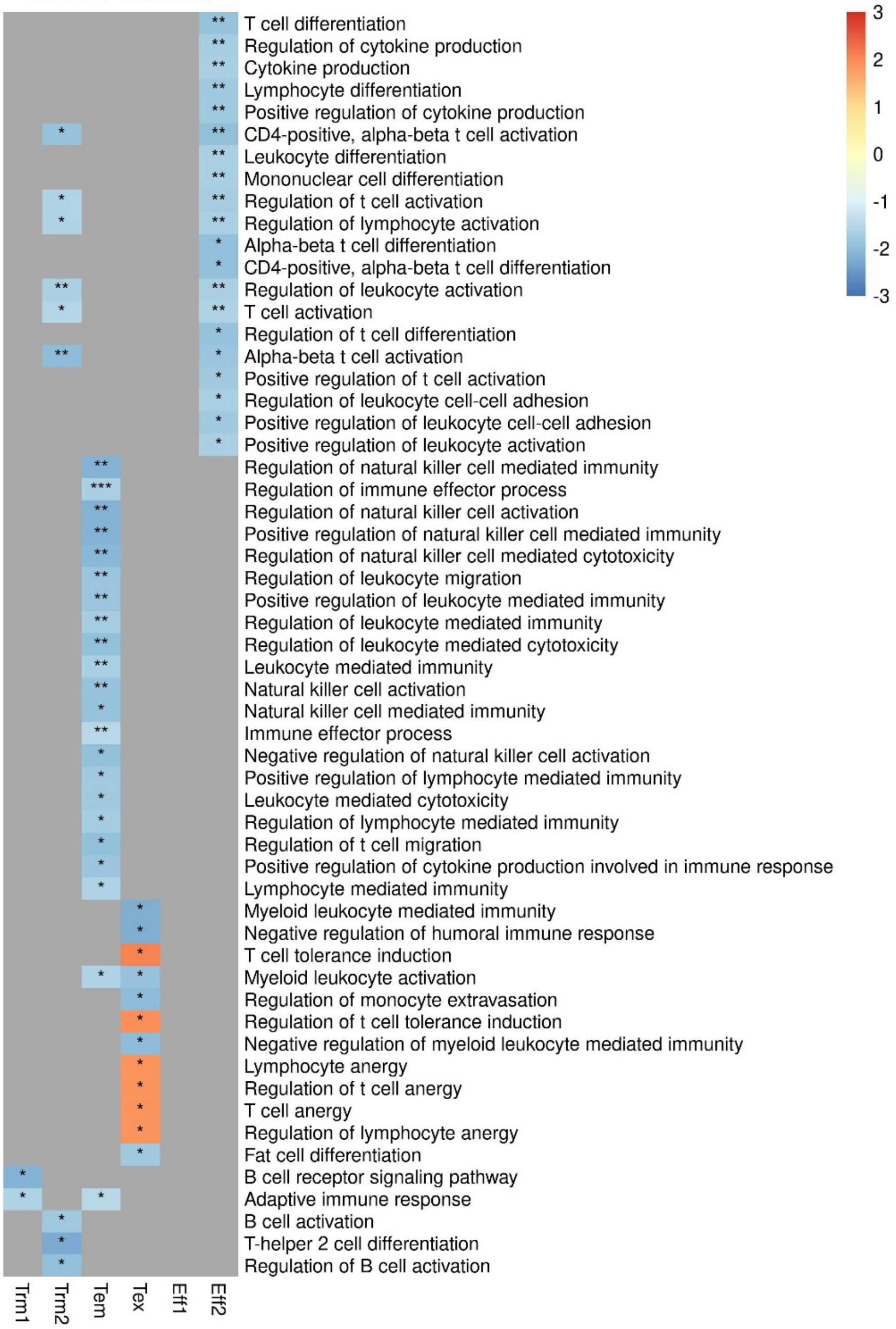

E **NK clusters**

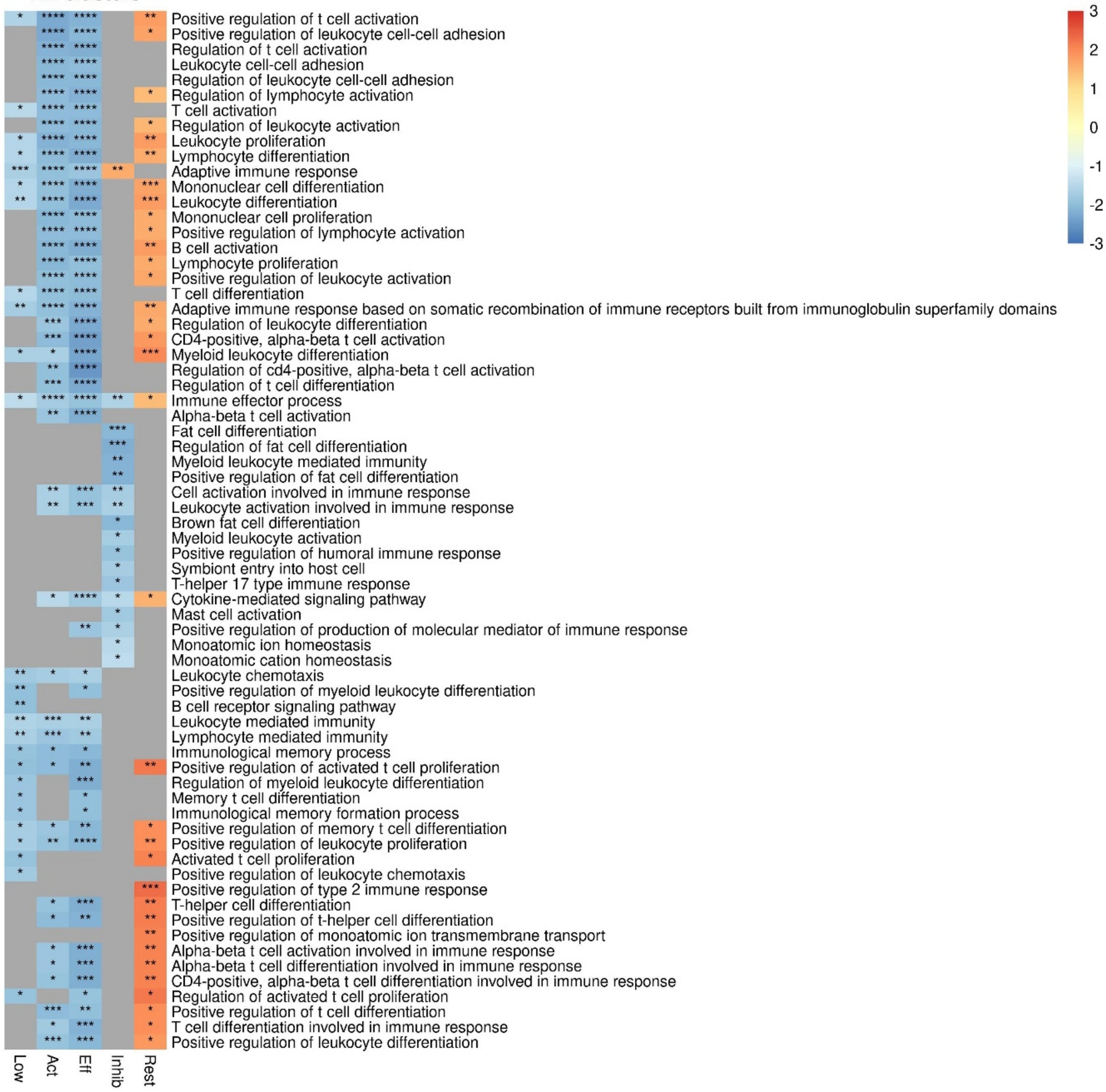

(A) Canonical marker gene expression across T/NK cell clusters

(B) Expression of functional marker genes across T/NK cell clusters

(C–E) Gene set enrichment analysis of $CD4^+$ T cells (C), $CD8^+$ T cells (D), and NK cells (E).

Heatmaps display normalized enrichment scores (NES) for selected Gene Ontology (GO) biological processes across each cell cluster. Red and blue colors indicate positive and negative enrichment, respectively. Statistical significance is indicated by asterisks (*), based on adjusted p-values.

*p < 0.05, **p < 0.01, ***p < 0.001, ****p < 0.0001 (adjusted p-values).

Supplementary Figure 3. Cell enrichment analysis of T and NK subsets from tumor tissue

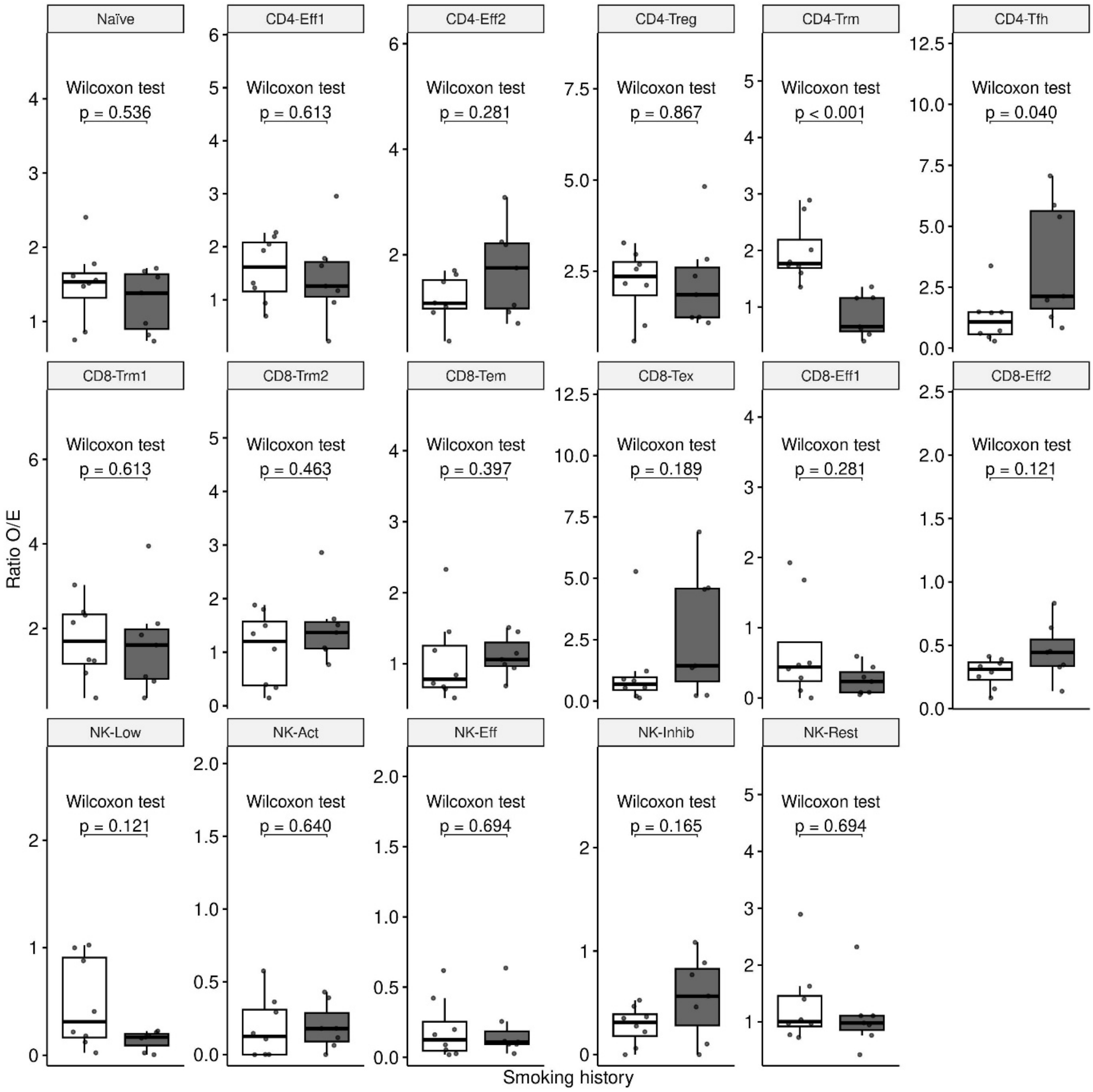

Supplementary Figure 4. CXCL13 expression is associated with immune checkpoint activation across cohorts and tissue validation

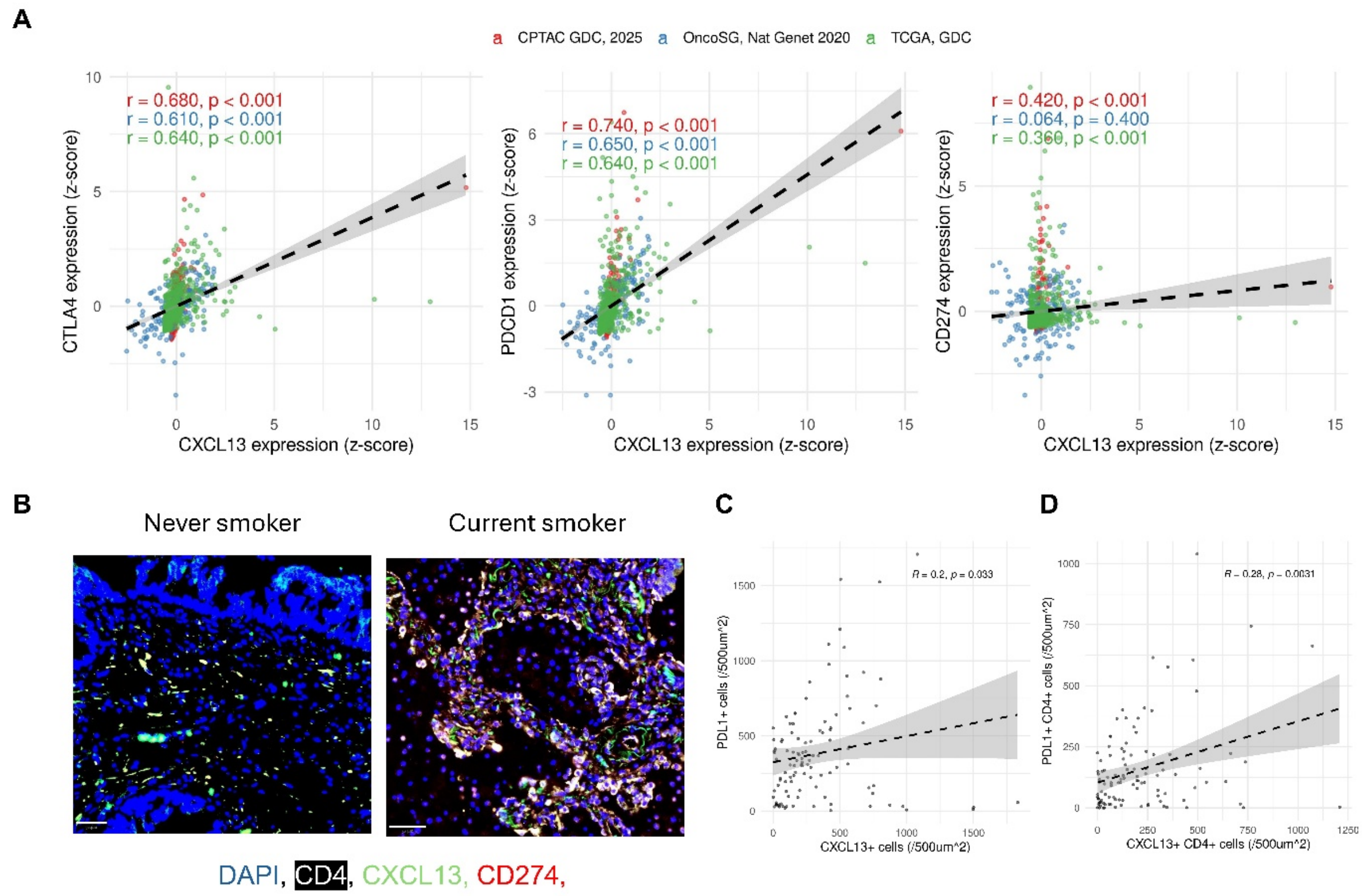

(A) Correlation between CXCL13 expression and immune checkpoint genes across independent cohorts (CPTAC, OncoSG, and TCGA). (A, left) CTLA4, (middle) PDCD1, and (right) CD274 expression are shown as a function of CXCL13 expression. Each dot represents a sample, and dashed lines indicate linear regression with 95% confidence intervals. Correlation coefficients (r) and p-values are shown.

(B) Representative immunofluorescence images showing CXCL13^{+} CD4^{+} T cells and PD-L1 (CD274) expression in tumor tissues (CXCL13, green; CD4, white; CD274, red; DAPI, blue; scale bar: 50 μm).

(C) Correlation between CXCL13^{+} cell density and PD-L1^{+} cell density. Scatter plot showing the relationship between CXCL13^{+} cells and PD-L1^{+} cells within the tumor microenvironment. Each dot represents an individual tumor sample. The dashed line indicates the linear regression fit, and the shaded area represents the 95% confidence interval. A weak but significant positive correlation was observed ($\rho = 0.20$, $p = 0.033$).

(D) Correlation between CXCL13^{+}CD4^{+} cell density and PD-L1^{+}CD4^{+} cell density. Scatter plot showing the relationship between CXCL13^{+}CD4^{+} cells and PD-L1^{+}CD4^{+} cells. Each dot represents an individual tumor sample. The dashed line indicates the linear regression fit, and the shaded area represents the 95% confidence interval. A weak but significant positive correlation was identified ($\rho = 0.28$, $p = 0.003$), suggesting an association between CXCL13-related CD4^{+} T-cell activity and PD-L1 expression within the tumor immune microenvironment.

Supplementary Figure 5. Smoking-associated alterations in CellChat-predicted cell–cell communication patterns in tumor and normal tissues.

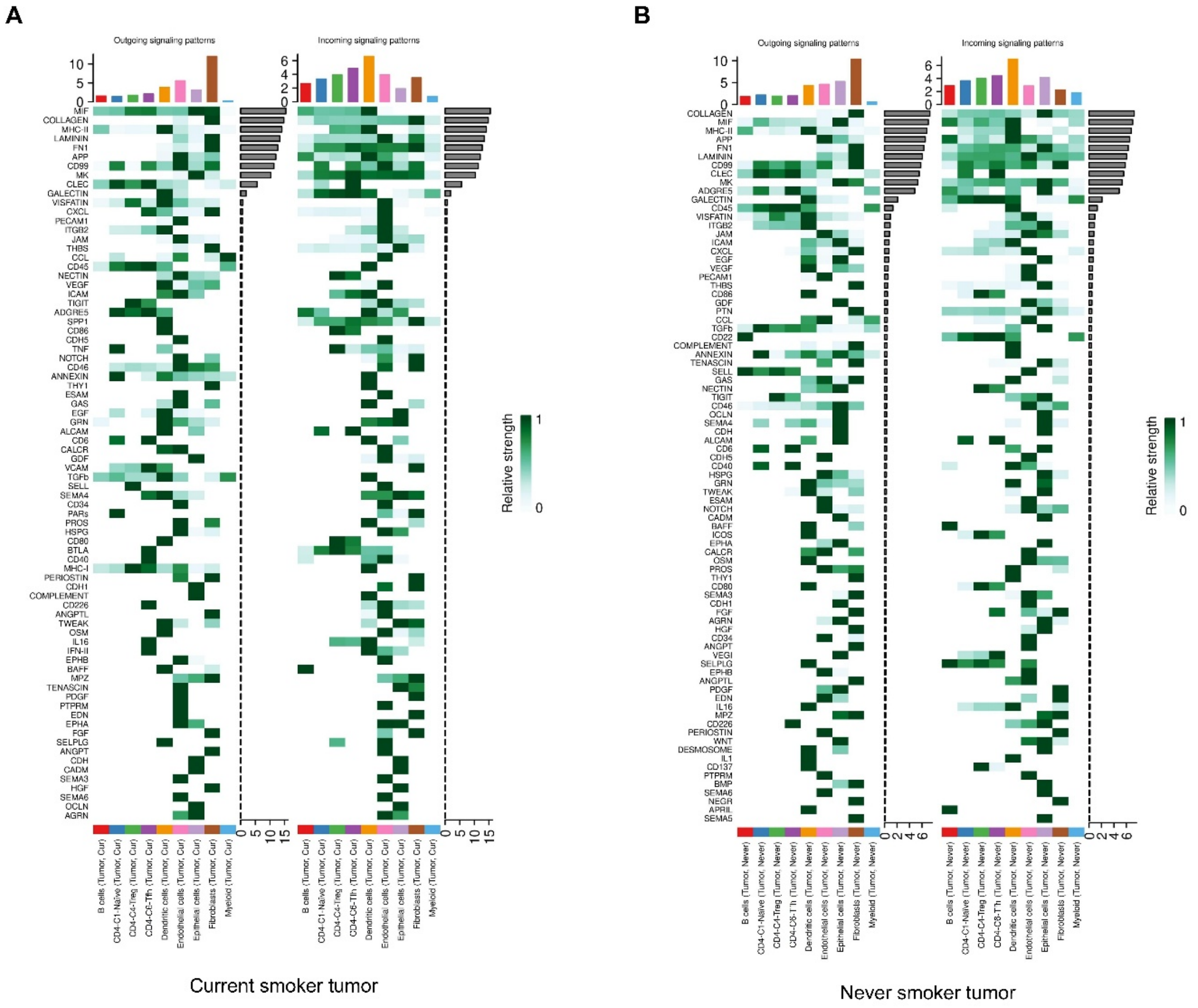

C

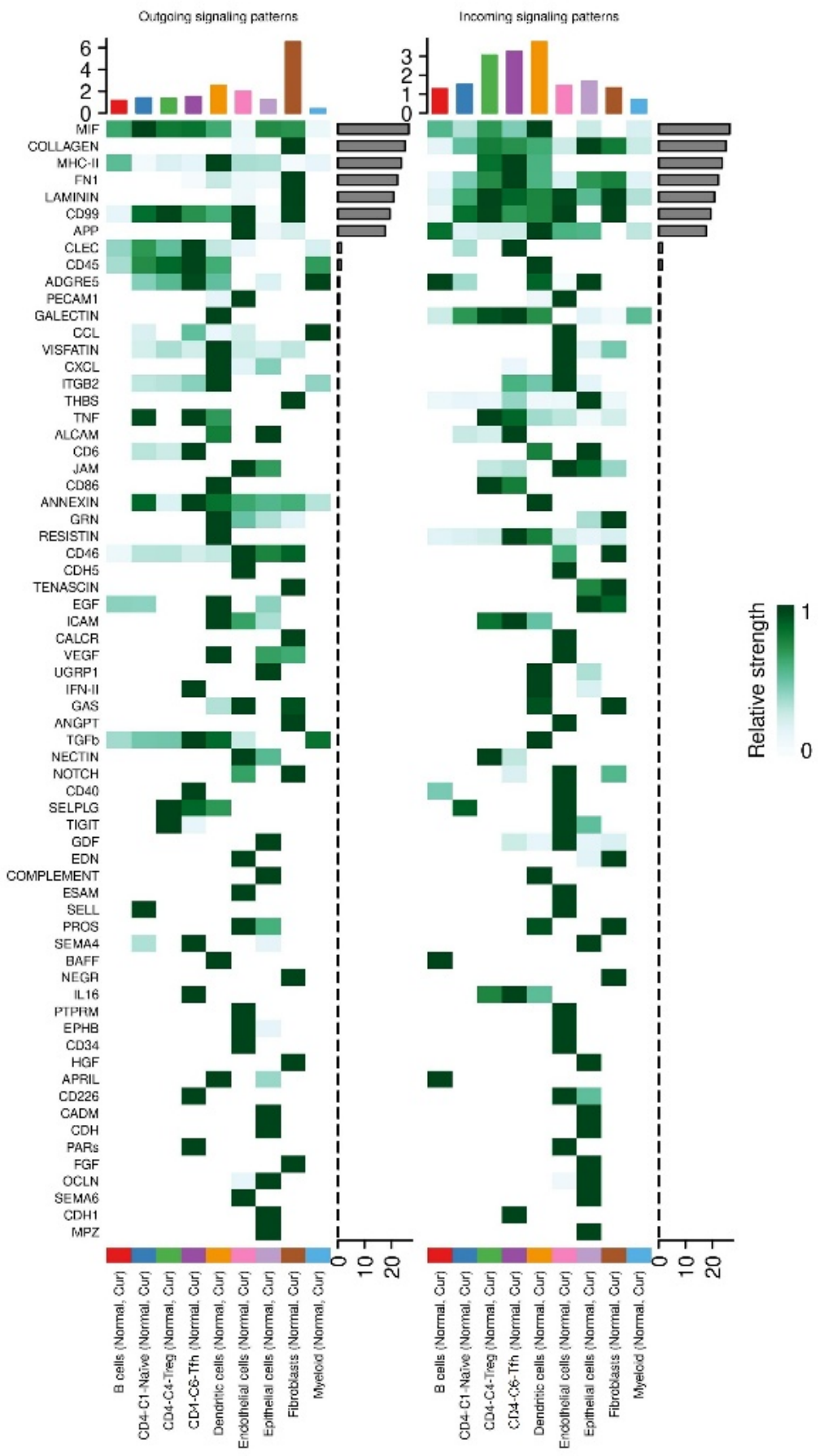

Current smoker normal

D

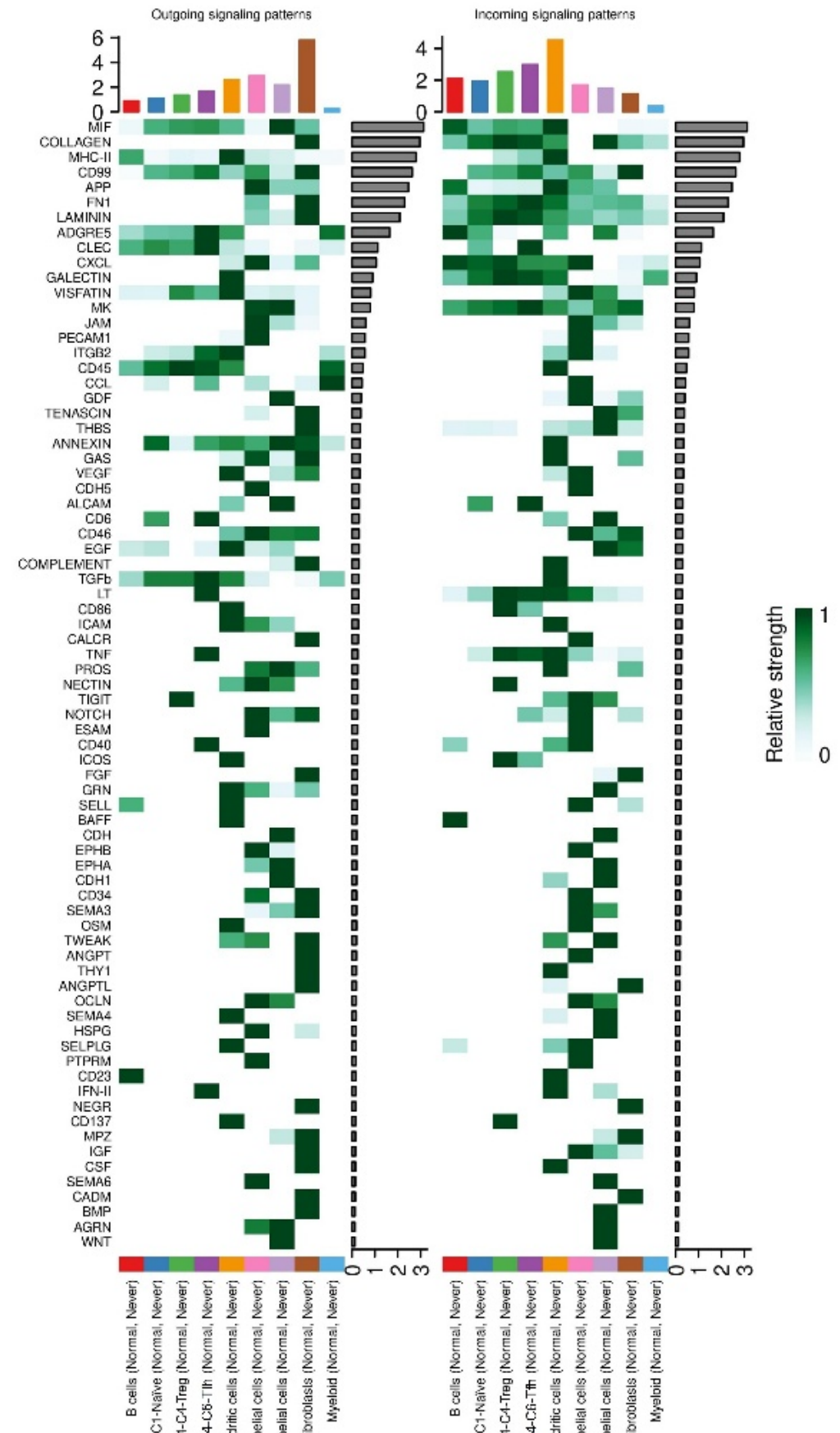

Never smoker normal

E

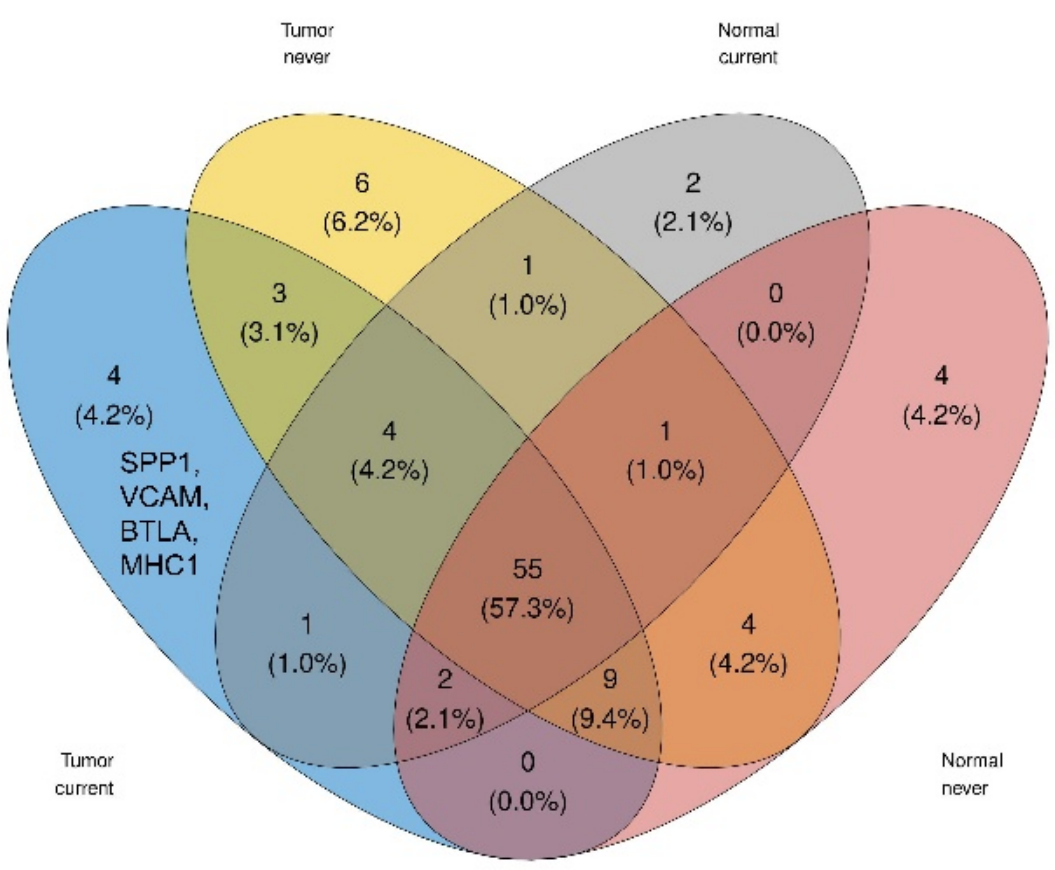

F

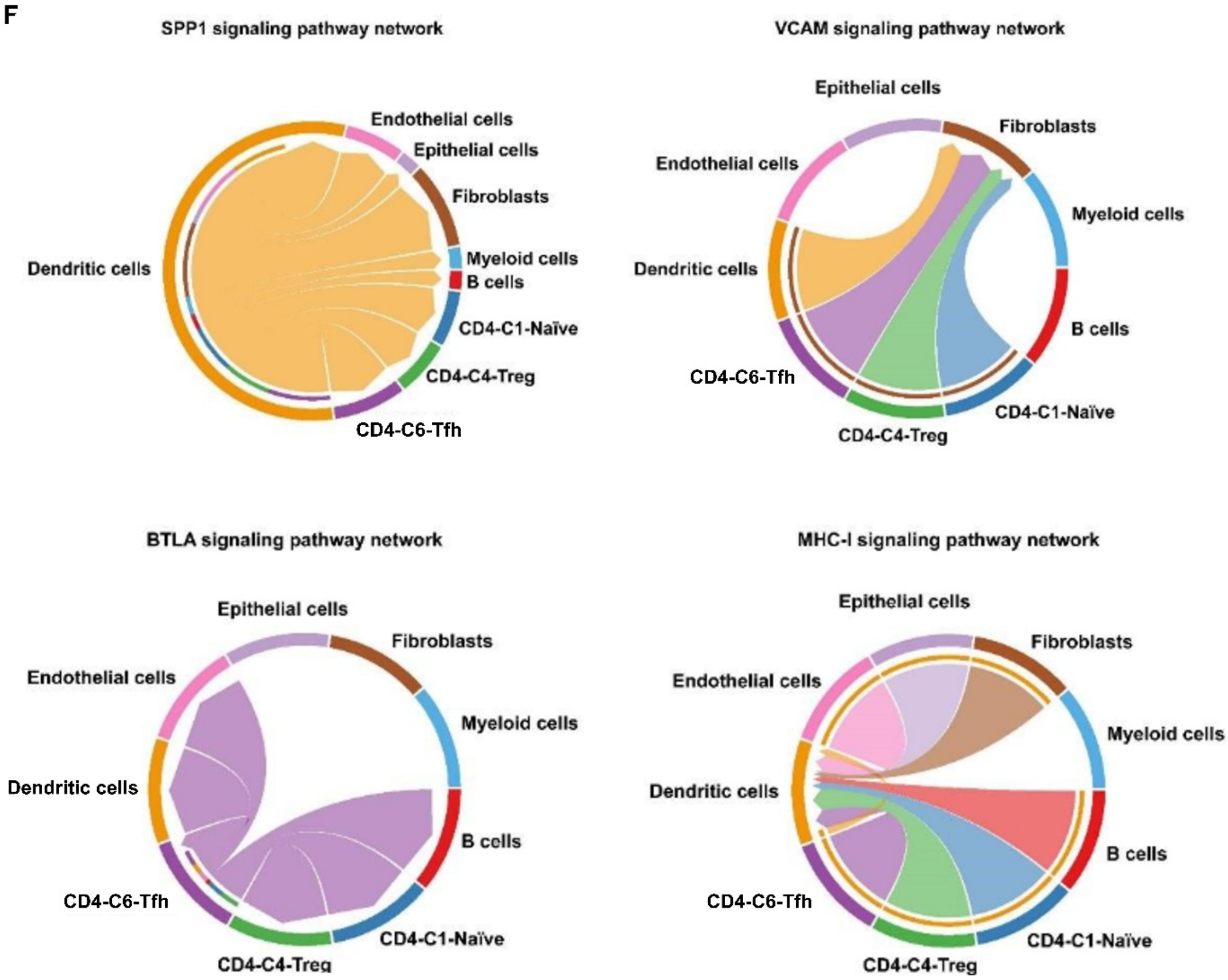
SPP1 signaling pathway network
Endothelial cells
Epithelial cells
Fibroblasts
Myeloid cells
B cells
CD4-C1-Naïve
CD4-C4-Treg
CD4-C6-Tfh
Dendritic cells
VCAM signaling pathway network
Epithelial cells
Fibroblasts
Endothelial cells
Myeloid cells
Dendritic cells
B cells
CD4-C6-Tfh
CD4-C1-Naïve
CD4-C4-Treg
BTLA signaling pathway network
Epithelial cells
Fibroblasts
Endothelial cells
Myeloid cells
Dendritic cells
B cells
CD4-C6-Tfh
CD4-C1-Naïve
CD4-C4-Treg
MHC-I signaling pathway network
Epithelial cells
Fibroblasts
Endothelial cells
Myeloid cells
Dendritic cells
B cells
CD4-C6-Tfh
CD4-C1-Naïve
CD4-C4-Treg

E

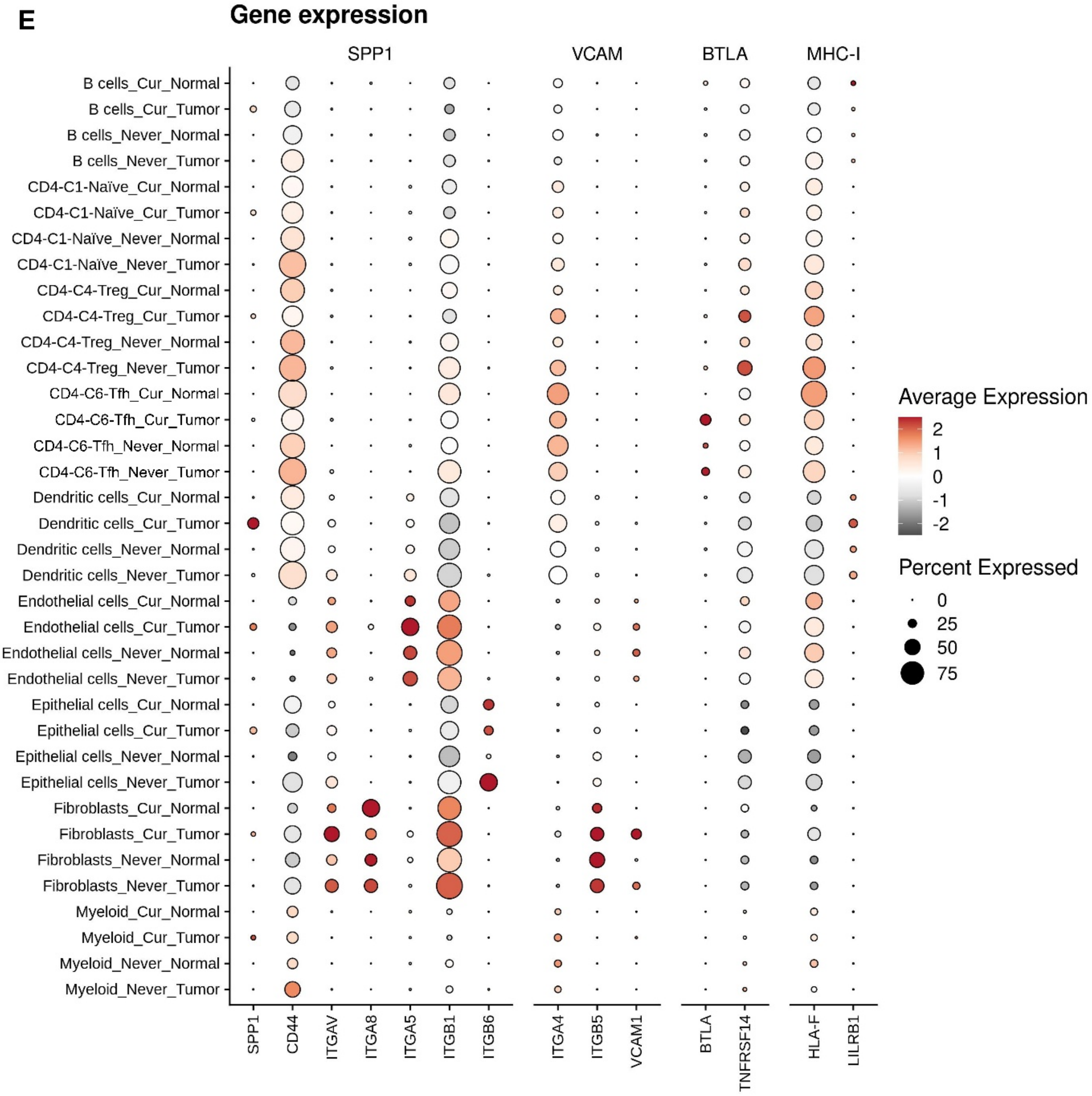

(A–B) Heatmaps showing outgoing (left) and incoming (right) signaling patterns across major cell populations in tumor tissues from current smokers (A) and never-smokers (B). (C–D) Heatmaps showing outgoing (left) and incoming (right) signaling patterns across major cell populations in normal tissues from current smokers (C) and never-smokers (D). Each row represents a signaling pathway, and each column represents a cell type. Color intensity indicates relative signaling strength. In tumor tissues, current smokers exhibit globally enhanced and diversified intercellular communication compared with never-smokers, whereas such differences are less pronounced in normal tissues.

(E) Venn diagram showing the overlap of CellChat-predicted signaling pathways across tumor and adjacent normal tissues stratified by smoking status. Each set represents signaling pathways inferred by CellChat under the corresponding tissue and smoking condition. A large proportion of predicted pathways was shared across

all conditions (57.3%), whereas SPP1, VCAM, BTLA, and MHC-I signaling were identified only among the predicted pathways in tumor tissues from current smokers.

(F) Chord diagrams illustrating CellChat-inferred communication networks for selected signaling pathways identified in tumors from current smokers. The diagrams show predicted SPP1-, VCAM-, BTLA-, and MHC-I-related communication across major cell populations, with chord width representing relative predicted interaction strength. SPP1- and VCAM-related signaling showed relatively prominent predicted communication between stromal populations, including fibroblasts and endothelial cells, and immune cell populations. BTLA-related signaling was predominantly predicted to involve $CD4^{+}$ follicular helper T (Tfh) cells. MHC-I-related signaling showed broadly distributed predicted communication across multiple immune and non-immune cell populations. These patterns represent computationally inferred communication and do not establish direct functional interactions.

(G) Dot plot showing expression of key ligand–receptor genes associated with the selected signaling pathways across cell populations stratified by smoking status and tissue type. Expression of genes related to SPP1, VCAM, BTLA, and MHC-I signaling is shown across major cell types in tumor and adjacent normal tissues from current and never-smokers. Dot size represents the proportion of expressing cells, and color intensity indicates average expression level. VCAM-related genes, including VCAM1 and integrins, were prominently expressed in stromal compartments, particularly fibroblasts and endothelial cells. These expression patterns are consistent with the CellChat-predicted VCAM-related signaling pattern but do not demonstrate functional intercellular communication.

Supplementary Figure 6. External validation of CXCL13-associated signaling gene expression across independent cohorts.

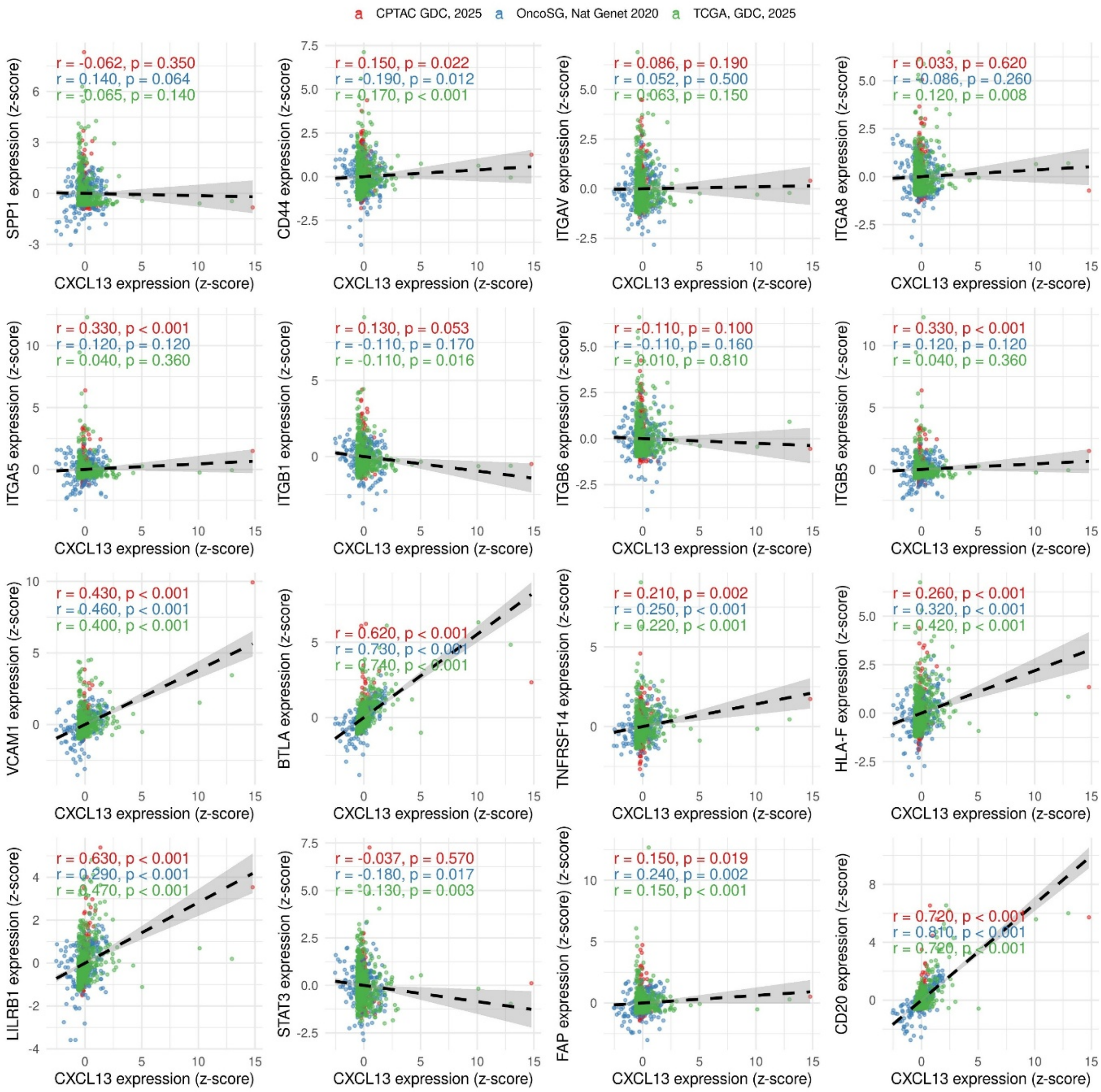

Scatter plots showing correlations between CXCL13 expression and genes related to SPP1, VCAM, BTLA, and MHC-I signaling pathways across three independent lung adenocarcinoma cohorts (CPTAC, OncoSG, and TCGA).

Correlation coefficients and corresponding p-values are indicated for each cohort. Overall, CXCL13 expression was positively correlated with VCAM1, FAP, and CD20 expression across multiple datasets, supporting a consistent association between Tfh-related signals and stromal and B-cell components.
